# Supplementary material for: Neuronal junctophilins recruit specific CaV and RyR isoforms to ER-PM junctions and functionally alter CaV2.1 and CaV2.2
Source: eLife. 2021 Mar 26;10:e64249. doi: 10.7554/eLife.64249 (PMC8046434; doi:10.7554/eLife.64249)
Supplement: Figure 2—source data 1. [file elife-64249-fig2-data1.docx]

**Figure 2D-G**

**Ca_V_1.2, Ca_V_2.1, Ca_V_2.2, Ca_V_3.1 vs JPH3 and JPH4**

**Pearson’s Coefficients**

| **Cell** | **Ca_V_1.2 vs** | | **Ca_V_2.1 vs** | | **Ca_V_2.2 vs** | | **Ca_V_3.1 vs** | |
| --- | --- | --- | --- | --- | --- | --- | --- | --- |
|  | JPH3 | JPH4 | JPH3 | JPH4 | JPH3 | JPH4 | JPH3 | JPH4 |
| 1 | 0.66 | 0.57 | 0.23 | 0.63 | 0.55 | 0.88 | 0.15 | 0.25 |
| 2 | 0.69 | 0.67 | 0.63 | 0.58 | 0.6 | 0.81 | 0.11 | 0.35 |
| 3 | 0.56 | 0.69 | 0.41 | 0.42 | 0.72 | 0.76 | 0.45 | 0.41 |
| 4 | 0.76 | 0.67 | 0.58 | 0.69 | 0.87 | 0.89 | 0.25 | 0.36 |
| 5 | 0.71 | 0.44 | 0.43 | 0.62 | 0.89 | 0.88 | 0.32 | 0.38 |
| 6 | 0.65 | 0.76 | 0.46 | 0.42 | 0.54 | 0.84 | 0.34 | 0.51 |
| 7 | 0.75 | 0.46 | 0.5 | 0.55 | 0.78 | 0.86 | 0.42 | 0.37 |
| 8 | 0.67 | 0.68 | 0.73 | 0.67 | 0.69 | 0.81 | 0.51 | 0.39 |
| 9 | 0.78 | 0.56 | 0.67 | 0.66 | 0.75 | 0.9 | 0.18 | 0.24 |
| 10 | 0.81 | 0.69 | 0.62 | 0.55 | 0.73 | 0.84 | 0.05 | 0.4 |
| 11 | 0.82 | 0.6 | 0.23 | 0.65 | 0.8 | 0.75 | 0.4 | 0.47 |
| 12 | 0.78 | 0.65 | 0.44 | 0.63 | 0.53 | 0.82 | 0.09 | 0.27 |
| 13 | 0.73 | 0.64 | 0.19 | 0.5 | 0.68 | 0.87 | 0.18 | 0.27 |
| 14 | 0.79 | 0.53 | 0.39 | 0.25 | 0.73 | 0.52 | 0.32 | 0.41 |
| 15 | 0.77 | 0.63 | 0.32 | 0.42 | 0.68 | 0.82 | 0.42 | 0.53 |
| 16 | 0.73 | 0.8 | 0.54 | 0.74 | 0.74 | 0.76 | 0.19 | 0.36 |
| 17 | 0.79 | 0.59 | 0.56 | 0.42 | 0.65 | 0.87 | 0.26 | 0.32 |
| 18 | 0.68 | 0.57 | 0.78 | 0.43 | 0.77 | 0.77 | 0.25 | 0.14 |
| 19 | 0.85 | 0.75 | 0.54 | 0.49 | 0.75 | 0.81 | 0.15 | 0.34 |
| 20 | 0.64 | 0.73 | 0.27 | 0.61 | 0.76 | 0.7 | 0.15 |  |
| 21 | 0.74 | 0.44 | 0.56 | 0.58 | 0.8 | 0.77 | 0.24 |  |
| 22 | 0.75 | 0.82 | 0.62 | 0.53 | 0.48 | 0.89 | 0.13 |  |
| 23 | 0.67 | 0.78 | 0.48 | 0.57 | 0.5 | 0.67 | 0.13 |  |
| 24 | 0.84 | 0.67 | 0.48 | 0.22 | 0.79 | 0.79 |  |  |
| 25 |  | 0.66 | 0.75 | 0.41 |  | 0.54 |  |  |
| 26 |  |  |  | 0.73 |  |  |  |  |
| 27 |  |  |  | 0.5 |  |  |  |  |
| 28 |  |  |  | 0.5 |  |  |  |  |

**Statistics**

**One-way ANOVA:** p < 0.0001

**Tukey's multiple comparisons test:**

| **Tukey's multiple comparisons test** | **Mean Diff.** | **95% CI of diff.** | **Significant?** | **Summary** | **Adjusted p Value** |
| --- | --- | --- | --- | --- | --- |
|  |  |  |  |  |  |
| [Ca_V_1.2 vs JPH3] vs [Ca_V_1.2 vs JPH4] | 0.09217 | -0.01047 to 0.1948 | No | ns | 0.1137 |
| [Ca_V_1.2 vs JPH3] vs [Ca_V_2.1 vsJPH3] | 0.2378 | 0.1351 to 0.3404 | Yes | **** | < 0.0001 |
| [Ca_V_1.2 vs JPH3] vs [Ca_V_2.1 vs JPH4] | 0.1995 | 0.09961 to 0.2994 | Yes | **** | < 0.0001 |
| [Ca_V_1.2 vs JPH3] vs [Ca_V_2.2 vs JPH3] | 0.0350 | -0.06868 to 0.1387 | No | ns | 0.9685 |
| [Ca_V_1.2 vs JPH3] vs [Ca_V_2.2 vs JPH4] | -0.05863 | -0.1613 to 0.04401 | No | ns | 0.6535 |
| [Ca_V_1.2 vs JPH3] vs [Ca_V_3.1 vs JPH3] | 0.4868 | 0.3820 to 0.5916 | Yes | **** | < 0.0001 |
| [Ca_V_1.2 vs JPH3] vs [Ca_V_3.1 vs JPH4] | 0.3779 | 0.2676 to 0.4881 | Yes | **** | < 0.0001 |
| [Ca_V_1.2 vs JPH4] vs [Ca_V_2.1 vs JPH3] | 0.1456 | 0.04401 to 0.2472 | Yes | *** | 0.0005 |
| [Ca_V_1.2 vs JPH4] vs [Ca_V_2.1 vs JPH4] | 0.1074 | 0.008528 to 0.2062 | Yes | * | 0.0229 |
| [Ca_V_1.2 vs JPH4] vs [Ca_V_2.2 vs JPH3] | -0.05717 | -0.1598 to 0.04547 | No | ns | 0.6823 |
| [Ca_V_1.2 vs JPH4] vs [Ca_V_2.2 vs JPH4] | -0.1508 | -0.2524 to -0.04921 | Yes | *** | 0.0003 |
| [Ca_V_1.2 vs JPH4] vs [Ca_V_3.1 vs JPH3] | 0.3946 | 0.2908 to 0.4984 | Yes | **** | < 0.0001 |
| [Ca_V_1.2 vs JPH4] vs [Ca_V_3.1 vs JPH4] | 0.2857 | 0.1764 to 0.3950 | Yes | **** | < 0.0001 |
| [Ca_V_2.1 vs JPH3] vs [Ca_V_2.1 vs JPH4] | -0.03824 | -0.1371 to 0.06059 | No | ns | 0.9350 |
| [Ca_V_2.1 vs JPH3] vs [Ca_V_2.2 vsJPH3] | -0.2028 | -0.3054 to -0.1001 | Yes | **** | < 0.0001 |
| [Ca_V_2.1 vs JPH3] vs [Ca_V_2.2 vs JPH4] | -0.2964 | -0.3980 to -0.1948 | Yes | **** | < 0.0001 |
| [Ca_V_2.1 vs JPH3] vs [Ca_V_3.1 vs JPH3] | 0.2490 | 0.1452 to 0.3528 | Yes | **** | < 0.0001 |
| [Ca_V_2.1 vs JPH3] vs [Ca_V_3.1 vs JPH4] | 0.1401 | 0.03077 to 0.2494 | Yes | ** | 0.0030 |
| [Ca_V_2.1 vs JPH4] vs [Ca_V_2.2 vs JPH3] | -0.1645 | -0.2644 to -0.06461 | Yes | **** | < 0.0001 |
| [Ca_V_2.1 vs JPH4] vs [Ca_V_2.2 vs JPH4] | -0.2582 | -0.3570 to -0.1593 | Yes | **** | < 0.0001 |
| [Ca_V_2.1 vs JPH4] vs [Ca_V_3.1 vs JPH3] | 0.2873 | 0.1862 to 0.3883 | Yes | **** | < 0.0001 |
| [Ca_V_2.1 vs JPH4] vs [Ca_V_3.1 vs JPH4] | 0.1783 | 0.07157 to 0.2851 | Yes | **** | < 0.0001 |
| [Ca_V_2.2 vs JPH3] vs [Ca_V_2.2 vs JPH4] | -0.09363 | -0.1963 to 0.009007 | No | ns | 0.1021 |
| [Ca_V_2.2 vs JPH3] vs [Ca_V_3.1 vs JPH3] | 0.4518 | 0.3470 to 0.5566 | Yes | **** | < 0.0001 |
| [Ca_V_2.2 vs JPH3] vs [Ca_V_3.1 vs JPH4] | 0.3429 | 0.2326 to 0.4531 | Yes | **** | < 0.0001 |
| [Ca_V_2.2 vs JPH4] vs [Ca_V_3.1 vs JPH3] | 0.5454 | 0.4416 to 0.6492 | Yes | **** | < 0.0001 |
| [Ca_V_2.2 vs JPH4] vs [Ca_V_3.1 vs JPH4] | 0.4365 | 0.3272 to 0.5458 | Yes | **** | < 0.0001 |
| [Ca_V_3.1 vs JPH3] vs [Ca_V_3.1 vs JPH4] | -0.1089 | -0.2203 to 0.002423 | No | ns | 0.0602 |
