# Supplementary material for: Neuronal junctophilins recruit specific CaV and RyR isoforms to ER-PM junctions and functionally alter CaV2.1 and CaV2.2
Source: eLife. 2021 Mar 26;10:e64249. doi: 10.7554/eLife.64249 (PMC8046434; doi:10.7554/eLife.64249)
Supplement: Figure 3—source data 1. [file elife-64249-fig3-data1.docx]

**Figure 3: Calcium currents**

**Fig. 3A**

**Ca_V_1.2 without JPHs**

**I_Peak_ (pA/pF)**

| **Test Potential (mV)** | **cell 1** | **cell 2** | **cell 3** | **cell 4** | **cell 5** | **cell 6** | **cell 7** | **cell 8** | **cell 9** | **cell 10** | **cell 11** | **cell 12** | **cell 13** | **cell 14** | **cell 15** | **cell 16** | **cell 17** |
| --- | --- | --- | --- | --- | --- | --- | --- | --- | --- | --- | --- | --- | --- | --- | --- | --- | --- |
| -20 | -0.48 | 0.50 | -0.49 | 1.02 | -0.64 | -0.35 | -0.20 | -0.76 | -0.27 | -0.61 | -0.48 | -0.77 | -0.31 | -0.22 | -0.55 | 1.39 | -0.54 |
| -10 | -1.61 | -1.26 | -1.12 | -2.10 | -0.80 | -0.92 | -0.37 | -1.90 | -0.29 | -3.09 | -1.71 | -3.42 | -1.00 | -0.38 | -2.16 | -3.32 | -1.74 |
| 0 | -5.25 | -3.81 | -2.88 | -5.65 | -1.11 | -2.99 | -0.66 | -8.59 | -0.62 | -12.58 | -6.16 | -19.30 | -3.96 | -1.23 | -8.74 | -12.98 | -6.62 |
| 10 | -12.41 | -8.54 | -5.45 | -11.64 | -2.15 | -7.44 | -1.49 | -25.78 | -1.51 | -33.14 | -15.20 | -65.14 | -10.08 | -2.79 | -23.87 | -31.09 | -15.85 |
| 20 | -21.05 | -14.76 | -8.30 | -18.07 | -3.51 | -12.80 | -2.41 | -49.12 | -2.72 | -51.48 | -24.97 | -84.07 | -17.92 | -4.68 | -41.51 | -45.91 | -25.52 |
| 30 | -26.89 | -18.84 | -10.54 | -22.06 | -4.92 | -16.94 | -3.50 | -62.72 | -4.25 | -54.94 | -29.36 | -75.18 | -24.00 | -6.53 | -50.46 | -48.80 | -29.31 |
| 40 | -26.32 | -19.63 | -10.70 | -21.81 | -5.64 | -18.08 | -4.00 | -60.75 | -5.25 | -48.12 | -26.81 | -57.51 | -25.40 | -7.16 | -47.42 | -40.73 | -27.05 |
| 50 | -21.72 | -16.94 | -9.05 | -17.08 | -5.45 | -16.64 | -4.09 | -49.52 | -5.50 | -36.13 | -20.41 | -41.48 | -22.54 | -6.69 | -37.02 | -29.15 | -21.72 |
| 60 | -15.18 | -13.25 | -7.61 | * | -4.37 | -12.94 | -3.56 | -36.13 | -4.90 | -24.60 | -13.40 | -28.00 | -16.99 | -5.13 | -25.28 | -17.98 | -15.56 |
| 70 | -9.30 | * | * | * | -3.06 | -8.86 | -2.81 | * | -3.83 | -15.26 | -7.80 | * | -11.27 | -3.20 | -15.40 | * | -9.73 |

* cell died

**I_700_ (pA/pF)**

| **Test Potential (mV)** | **cell 1** | **cell 2** | **cell 3** | **cell 4** | **cell 5** | **cell 6** | **cell 7** | **cell 8** | **cell 9** | **cell 10** | **cell 11** | **cell 12** | **cell 13** | **cell 14** | **cell 15** | **cell 16** | **cell 17** |
| --- | --- | --- | --- | --- | --- | --- | --- | --- | --- | --- | --- | --- | --- | --- | --- | --- | --- |
| 0 | -2.961 | -2.351 | -1.279 | -2.666 | -0.395 | -1.734 | -0.401 | -4.655 | -0.297 | -3.250 | -3.029 | -5.429 | -2.220 | -0.646 | -4.710 | -4.584 | -2.846 |
| 10 | -4.089 | -3.673 | -2.217 | -4.453 | -0.686 | -2.906 | -0.653 | -7.330 | -0.621 | -3.127 | -2.992 | -6.001 | -3.390 | -0.931 | -5.882 | -5.111 | -3.262 |
| 20 | -4.406 | -5.072 | -3.127 | -4.928 | -0.747 | -3.417 | -0.872 | -7.531 | -0.888 | -2.114 | -2.096 | -4.717 | -3.615 | -0.950 | -4.588 | -4.648 | -2.842 |
| 30 | -4.289 | -5.250 | -3.410 | -5.084 | -0.843 | -3.280 | -1.034 | -5.838 | -1.032 | -1.214 | -1.460 | -3.516 | -3.547 | -0.951 | -3.271 | -3.703 | -2.504 |
| 40 | -3.669 | -5.559 | -3.389 | -3.878 | -0.862 | -3.071 | -1.105 | -3.851 | -1.229 | -0.551 | -1.192 | -2.428 | -3.203 | -0.946 | -2.580 | -2.867 | -2.225 |
| 50 | -3.214 | -3.416 | -3.128 | -3.795 | -0.876 | -3.086 | -1.296 | -1.749 | -1.416 | -0.008 | -1.032 | -1.285 | -3.066 | -0.789 | -2.162 | -1.841 | -1.902 |
| 60 | -2.706 | -2.184 | -2.732 | * | -0.865 | -2.876 | -1.406 | -1.552 | -1.434 | -0.024 | -0.828 | -0.708 | -2.543 | -0.770 | -1.885 | -0.636 | -1.638 |
| 70 | -2.214 | * | * | * | -0.685 | -2.369 | -1.320 | * | -1.309 | -0.056 | -0.567 | * | -1.420 | -0.465 | -1.554 | * | -1.136 |

* cell died

**Fig 3A**

**Ca_V_1.2 with JPH3**

**I_Peak_ (pA/pF)**

| **Test Potential (mV)** | **cell 1** | **cell 2** | **cell 3** | **cell 4** | **cell 5** | **cell 6** | **cell 7** | **cell 8** | **cell 9** | **cell 10** | **cell 11** | **cell 12** | **cell 13** |
| --- | --- | --- | --- | --- | --- | --- | --- | --- | --- | --- | --- | --- | --- |
| -20 | -0.22 | -0.28 | -0.67 | -1.02 | -0.31 | 0.97 | 0.51 | -0.43 | 0.66 | -0.56 | 0.90 | -0.42 | -0.81 |
| -10 | -0.83 | -0.59 | -2.97 | -5.11 | -0.71 | 1.33 | -0.78 | -1.10 | -1.42 | -2.05 | -4.51 | -1.35 | -2.90 |
| 0 | -2.50 | -1.87 | -9.67 | -18.29 | -1.82 | -3.67 | -2.46 | -3.68 | -5.15 | -6.53 | -19.86 | -4.63 | -10.05 |
| 10 | -5.87 | -4.75 | -20.85 | -42.24 | -4.16 | -9.19 | -6.39 | -8.53 | -13.19 | -14.73 | -52.20 | -12.66 | -22.32 |
| 20 | -9.95 | -8.44 | -31.66 | -63.97 | -6.69 | -16.23 | -12.18 | -14.62 | -23.34 | -23.43 | -79.95 | -21.94 | -35.27 |
| 30 | -13.15 | -11.84 | -35.96 | -70.03 | -8.25 | -20.83 | -18.19 | -18.42 | -29.63 | -27.92 | -86.86 | -28.45 | -41.04 |
| 40 | -14.40 | -13.05 | -32.69 | -61.61 | -7.95 | -20.98 | -21.01 | -17.76 | -29.00 | -26.45 | -75.27 | -28.71 | -39.16 |
| 50 | -13.27 | -12.09 | -25.16 | -46.69 | -6.42 | -17.08 | -19.82 | -14.08 | -24.22 | -21.16 | -58.51 | -24.36 | -32.14 |
| 60 | -10.66 | -9.68 | -16.85 | -31.48 | -4.19 | -11.97 | -16.21 | -9.58 | -17.51 | -14.95 | -40.72 | -18.17 | -22.72 |
| 70 | -7.42 | -6.23 | -9.91 | -18.79 | * | -7.32 | -11.67 | -5.63 | * | -9.15 | -25.22 | -11.90 | -14.25 |

* cell died

**I_700_ (pA/pF)**

| **Test Potential (mV)** | **cell 1** | **cell 2** | **cell 3** | **cell 4** | **cell 5** | **cell 6** | **cell 7** | **cell 8** | **cell 9** | **cell 10** | **cell 11** | **cell 12** | **cell 13** |
| --- | --- | --- | --- | --- | --- | --- | --- | --- | --- | --- | --- | --- | --- |
| 0 | -1.585 | -1.060 | -4.488 | -9.152 | -0.987 | -2.002 | -1.625 | -1.904 | -2.872 | -3.640 | -8.186 | -2.821 | -4.200 |
| 10 | -2.730 | -2.004 | -4.499 | -10.520 | -1.095 | -2.392 | -2.834 | -2.366 | -3.731 | -4.323 | -8.288 | -3.992 | -4.419 |
| 20 | -3.525 | -2.482 | -3.298 | -8.552 | -0.991 | -1.987 | -3.801 | -1.990 | -3.265 | -3.668 | -6.280 | -4.005 | -3.482 |
| 30 | -3.828 | -2.483 | -2.535 | -6.685 | -0.689 | -1.524 | -3.957 | -1.406 | -2.901 | -2.964 | -4.689 | -3.136 | -2.774 |
| 40 | -3.788 | -2.297 | -2.102 | -5.551 | -0.572 | -1.208 | -3.909 | -1.139 | -2.343 | -2.475 | -3.742 | -2.710 | -2.168 |
| 50 | -3.686 | -2.224 | -1.674 | -4.910 | -0.200 | -0.975 | -3.754 | -0.954 | -2.035 | -2.195 | -3.141 | -2.424 | -1.775 |
| 60 | -3.400 | -1.902 | -1.238 | -4.325 | -0.215 | -0.862 | -3.486 | -0.821 | -1.980 | -1.987 | -2.644 | -2.299 | -1.235 |
| 70 | -2.650 | -1.221 | -0.867 | -3.385 | * | -0.631 | -3.189 | -0.500 | * | -1.457 | -1.870 | -1.920 | -0.756 |

* cell died

**Fig 3A Ca_V_1.2 with JPH4**

**I_Peak_ (pA/pF)**

| **Test Potential (mV)** | **cell 1** | **cell 2** | **cell 3** | **cell 4** | **cell 5** | **cell 6** | **cell 7** | **cell 8** | **cell 9** | **cell 10** | **cell 11** | **cell 12** |
| --- | --- | --- | --- | --- | --- | --- | --- | --- | --- | --- | --- | --- |
| -20 | -1.10 | -0.37 | -0.23 | -1.23 | -0.28 | -0.48 | -0.27 | -0.36 | -0.53 | -0.44 | -0.43 | -0.26 |
| -10 | -6.02 | -0.66 | -0.27 | -5.83 | -1.65 | -1.50 | -1.24 | -0.63 | -0.97 | -1.10 | -2.12 | -0.37 |
| 0 | -25.05 | -1.57 | -0.81 | -22.83 | -4.79 | -5.06 | -4.43 | -1.26 | -2.36 | -3.85 | -10.90 | -1.12 |
| 10 | -62.95 | -3.03 | -2.15 | -56.62 | -12.37 | -12.22 | -11.80 | -2.66 | -5.27 | -9.68 | -32.74 | -3.16 |
| 20 | -95.69 | -4.48 | -4.23 | -87.59 | -19.74 | -20.50 | -20.66 | -4.50 | -8.03 | -17.23 | -36.73 | -5.56 |
| 30 | -101.38 | -5.48 | -5.57 | -94.50 | -23.90 | -25.16 | -26.36 | -5.65 | -10.41 | -22.66 | -25.80 | -7.33 |
| 40 | -87.16 | -5.55 | -6.42 | -83.38 | -22.22 | -24.58 | -25.59 | -6.31 | -10.51 | -23.55 | -16.44 | -7.68 |
| 50 | -65.87 | -4.32 | -6.12 | -63.93 | -17.39 | -20.27 | -21.16 | -5.90 | -8.91 | -20.61 | -11.75 | -6.77 |
| 60 | -44.09 | -3.25 | -5.01 | -43.94 | -11.56 | -14.90 | -15.40 | -4.67 | -6.66 | -15.80 | -8.72 | -5.19 |
| 70 | -26.86 | -1.95 | -3.60 | -27.29 | -6.74 | -9.70 | -9.71 | -3.22 | -4.52 | -10.63 | -6.18 | -3.23 |

**I_700_ (pA/pF)**

| **Test Potential (mV)** | **cell 1** | **cell 2** | **cell 3** | **cell 4** | **cell 5** | **cell 6** | **cell 7** | **cell 8** | **cell 9** | **cell 10** | **cell 11** | **cell 12** |
| --- | --- | --- | --- | --- | --- | --- | --- | --- | --- | --- | --- | --- |
| 0 | -9.534 | -1.041 | -0.474 | -8.684 | -2.041 | -2.474 | -2.134 | -0.688 | -1.257 | -2.000 | -2.284 | -0.569 |
| 10 | -8.453 | -1.064 | -0.863 | -7.958 | -2.687 | -3.157 | -3.215 | -1.123 | -1.751 | -2.868 | -2.072 | -0.952 |
| 20 | -5.196 | -0.932 | -0.866 | -5.511 | -1.711 | -3.144 | -3.342 | -1.448 | -2.049 | -2.847 | -1.500 | -1.052 |
| 30 | -3.366 | -1.416 | -0.899 | -2.533 | -1.166 | -2.911 | -3.150 | -1.442 | -2.116 | -2.482 | -1.113 | -0.886 |
| 40 | -2.479 | -1.130 | -0.948 | -2.241 | -1.006 | -2.654 | -2.456 | -1.480 | -2.011 | -2.154 | -0.911 | -0.894 |
| 50 | -2.086 | -0.835 | -0.957 | -2.039 | -0.886 | -2.412 | -1.800 | -1.403 | -1.895 | -1.965 | -0.652 | -0.909 |
| 60 | -1.855 | -0.691 | -0.967 | -1.816 | -0.759 | -2.143 | -0.610 | -1.416 | -1.722 | -1.738 | -0.414 | -0.714 |
| 70 | -1.587 | -0.420 | -0.818 | -1.724 | -0.546 | -1.679 | -0.526 | -1.133 | -1.453 | -1.461 | -0.189 | -0.418 |

**Fig 3B**  **Ca_V_2.1 without JPHs**

**I_Peak_ (pA/pF)**

| **Test Potential (mV)** | **cell 1** | **cell 2** | **cell 3** | **cell 4** | **cell 5** | **cell 6** | **cell 7** | **cell 8** | **cell 9** |
| --- | --- | --- | --- | --- | --- | --- | --- | --- | --- |
| -10 | -0.35 | -0.27 | -0.32 | -0.11 | -0.65 | -0.37 | -0.24 | -0.51 | -0.36 |
| 0 | -0.38 | -0.60 | -0.55 | -0.78 | -2.72 | -0.73 | -1.13 | -1.14 | -1.07 |
| 10 | -0.63 | -2.72 | -2.38 | -3.44 | -18.68 | -2.68 | -5.29 | -5.79 | -5.21 |
| 20 | -1.54 | -12.61 | -7.29 | -17.94 | -70.15 | -12.87 | -26.51 | -20.97 | -23.97 |
| 30 | -3.93 | -26.15 | -9.24 | -35.97 | -65.62 | -25.88 | -47.40 | -30.63 | -42.26 |
| 40 | -4.90 | -25.29 | -7.07 | -33.01 | -48.93 | -24.07 | -41.92 | -25.89 | -37.67 |
| 50 | -4.11 | -17.85 | -4.55 | -23.52 | -33.66 | -17.05 | -30.29 | -18.14 | -26.54 |
| 60 | -2.83 | -11.31 | -2.28 | -14.42 | -20.36 | -10.29 | -19.18 | -11.04 | -16.30 |
| 70 | -1.63 | -6.26 | -1.18 | -7.81 | -11.36 | -5.45 | -10.94 | -6.05 | -9.01 |

**I_700_ (pA/pF)**

| **Test Potential (mV)** | **cell 1** | **cell 2** | **cell 3** | **cell 4** | **cell 5** | **cell 6** | **cell 7** | **cell 8** | **cell 9** |
| --- | --- | --- | --- | --- | --- | --- | --- | --- | --- |
| **0** | -0.178 | -0.286 | -0.247 | -0.350 | -1.257 | -0.233 | -0.358 | -0.339 | -0.432 |
| **10** | -0.131 | -1.202 | -0.845 | -1.502 | -6.089 | -1.273 | -1.398 | -1.014 | -1.571 |
| **20** | -0.341 | -2.536 | -1.298 | -4.119 | -10.690 | -2.975 | -3.035 | -1.369 | -3.092 |
| **30** | -0.539 | -2.883 | -1.484 | -4.739 | -8.582 | -3.353 | -3.248 | -1.514 | -3.237 |
| **40** | -0.490 | -2.799 | -1.298 | -4.484 | -6.067 | -3.515 | -3.037 | -1.343 | -3.242 |
| **50** | -0.345 | -2.341 | -1.065 | -3.878 | -4.160 | -2.694 | -2.685 | -1.145 | -2.803 |
| **60** | -0.218 | -1.532 | -0.220 | -2.460 | -2.605 | -1.871 | -2.083 | -0.889 | -2.106 |
| **70** | -0.160 | -0.916 | -0.111 | -1.248 | -1.495 | -1.115 | -1.274 | -0.585 | -1.364 |

**Fig 3B Ca_V_2.1 with JPH3**

**I_Peak_ (pA/pF)**

| **Test Potential (mV)** | **cell 1** | **cell 2** | **cell 3** | **cell 4** | **cell 5** | **cell 6** | **cell 7** | **cell 8** | **cell 9** |
| --- | --- | --- | --- | --- | --- | --- | --- | --- | --- |
| -10 | -0.17 | -0.12 | -0.11 | -0.27 | -0.19 | -0.17 | -0.18 | -0.24 | -0.13 |
| 0 | -0.48 | -0.15 | -0.14 | -1.52 | -0.51 | -0.29 | -0.61 | -0.29 | -0.53 |
| 10 | -2.04 | -0.62 | -1.30 | -9.18 | -2.50 | -3.65 | -2.84 | -3.73 | -2.44 |
| 20 | -12.06 | -2.40 | -6.28 | -48.03 | -8.19 | -18.76 | -12.37 | -18.81 | -12.44 |
| 30 | -25.97 | -5.94 | -10.40 | -73.20 | -10.06 | -41.41 | -21.73 | -41.44 | -22.05 |
| 40 | -22.70 | -6.48 | -8.82 | -59.94 | -7.62 | -38.64 | -19.06 | -38.70 | -19.18 |
| 50 | -16.06 | -4.53 | -5.99 | -39.94 | -5.08 | -26.76 | -13.32 | -26.83 | -13.50 |
| 60 | -10.01 | -2.49 | -3.52 | -23.16 | -2.89 | -16.13 | -8.07 | -16.20 | -8.46 |
| 70 | -5.56 | -1.24 | -2.18 | -11.33 | -1.33 | -8.82 | -4.41 | -8.88 | -4.84 |

**I_700_ (pA/pF)**

| **Test Potential (mV)** | **cell 1** | **cell 2** | **cell 3** | **cell 4** | **cell 5** | **cell 6** | **cell 7** | **cell 8** | **cell 9** |
| --- | --- | --- | --- | --- | --- | --- | --- | --- | --- |
| 0 | -0.105 | -0.257 | -0.083 | -0.069 | -0.478 | -0.325 | -0.156 | -0.308 | -0.284 |
| 10 | -0.305 | -0.954 | -0.325 | -0.661 | -2.745 | -1.701 | -2.123 | -1.335 | -1.232 |
| 20 | -1.098 | -3.416 | -1.130 | -2.143 | -6.538 | -4.122 | -8.607 | -3.541 | -4.195 |
| 30 | -1.544 | -4.884 | -1.818 | -2.599 | -8.124 | -3.987 | -12.509 | -4.608 | -5.717 |
| 40 | -1.469 | -4.798 | -1.708 | -2.212 | -7.120 | -2.958 | -11.484 | -4.314 | -5.153 |
| 50 | -1.154 | -4.518 | -1.103 | -1.363 | -5.323 | -1.816 | -8.616 | -3.371 | -3.879 |
| 60 | -0.744 | -3.156 | -0.356 | -0.532 | -3.137 | -0.991 | -5.329 | -2.161 | -2.579 |
| 70 | -0.390 | -2.024 | -0.065 | -0.157 | -0.597 | -0.292 | -2.841 | -1.102 | -1.616 |

**Fig 3B Ca_V_2.1 with JPH4**

**I_Peak_ (pA/pF)**

| **Test Potential (mV)** | **cell 1** | **cell 2** | **cell 3** | **cell 4** | **cell 5** | **cell 6** | **cell 7** | **cell 8** | **cell 9** |
| --- | --- | --- | --- | --- | --- | --- | --- | --- | --- |
| -10 | -0.37 | -0.21 | -0.96 | -0.20 | -0.22 | -0.52 | -0.19 | -0.30 | -0.14 |
| 0 | -0.51 | -0.57 | -5.05 | -0.26 | -0.44 | -0.80 | -0.56 | -0.83 | -0.35 |
| 10 | -1.57 | -2.94 | -26.17 | -0.93 | -2.18 | -4.19 | -2.49 | -3.39 | -2.14 |
| 20 | -6.29 | -15.96 | -63.48 | -4.39 | -7.26 | -15.93 | -11.41 | -11.63 | -8.88 |
| 30 | -12.21 | -29.79 | -61.61 | -10.03 | -10.78 | -26.59 | -22.86 | -16.78 | -15.10 |
| 40 | -11.62 | -25.24 | -44.39 | -10.26 | -9.32 | -23.22 | -20.40 | -14.26 | -13.02 |
| 50 | -8.31 | -16.77 | -28.33 | -7.53 | -6.30 | -16.36 | -13.63 | -9.58 | -8.63 |
| 60 | -5.10 | -9.84 | -16.13 | -4.31 | -3.71 | -9.43 | -7.63 | -5.67 | -4.81 |
| 70 | -2.64 | -5.07 | -8.47 | -2.34 | -2.05 | -5.49 | -3.24 | -3.11 | -2.15 |

**I_700_ (pA/pF)**

| **Test Potential (mV)** | **cell 1** | **cell 2** | **cell 3** | **cell 4** | **cell 5** | **cell 6** | **cell 7** | **cell 8** | **cell 9** |
| --- | --- | --- | --- | --- | --- | --- | --- | --- | --- |
| 0 | -0.398 | -0.470 | -3.555 | -0.214 | -0.359 | -0.647 | -0.454 | -0.680 | -0.274 |
| 10 | -1.121 | -2.527 | -18.488 | -0.732 | -1.773 | -3.374 | -1.963 | -2.942 | -1.710 |
| 20 | -4.975 | -13.495 | -30.234 | -3.749 | -5.764 | -13.553 | -9.844 | -9.704 | -6.979 |
| 30 | -9.136 | -19.178 | -25.313 | -7.783 | -6.814 | -18.902 | -15.668 | -12.188 | -9.803 |
| 40 | -8.373 | -15.217 | -17.992 | -7.143 | -5.390 | -15.537 | -12.874 | -9.805 | -7.709 |
| 50 | -6.037 | -10.268 | -11.892 | -5.136 | -3.682 | -11.074 | -8.742 | -6.617 | -5.049 |
| 60 | -3.667 | -6.262 | -7.182 | -2.812 | -2.108 | -6.438 | -4.860 | -4.017 | -2.270 |
| 70 | -2.006 | -3.245 | -3.886 | -1.395 | -1.110 | -3.636 | -2.042 | -2.261 | -0.839 |

**Fig 3C Ca_V_2.2 without JPHs**

**I_Peak_ (pA/pF)**

| **Test Potential (mV)** | **cell 1** | **cell 2** | **cell 3** | **cell 4** | **cell 5** | **cell 6** | **cell 7** | **cell 8** | **cell 9** | **cell 10** | **cell 11** | **cell 12** | **cell 13** | **cell 14** |
| --- | --- | --- | --- | --- | --- | --- | --- | --- | --- | --- | --- | --- | --- | --- |
| -10 | -0.68 | -0.27 | -0.38 | -0.34 | -0.58 | -0.84 | -0.38 | -0.50 | -0.50 | -0.40 | -0.70 | -0.35 | -0.38 | -0.41 |
| 0 | -1.05 | -0.77 | -0.85 | -0.76 | -1.28 | -1.84 | -0.68 | -1.02 | -1.31 | -0.62 | -1.09 | -0.65 | -1.79 | -0.83 |
| 10 | -3.19 | -2.95 | -3.29 | -2.73 | -6.38 | -9.25 | -1.97 | -4.44 | -6.88 | -1.31 | -4.08 | -3.41 | -4.91 | -2.83 |
| 20 | -11.35 | -13.26 | -17.39 | -9.11 | -28.20 | -46.24 | -7.88 | -18.65 | -64.21 | -4.77 | -14.69 | -14.06 | -25.07 | -12.02 |
| 30 | -22.92 | -31.07 | -55.22 | -16.47 | -52.06 | -81.51 | -18.95 | -39.35 | -107.26 | -12.14 | -30.97 | -34.40 | -63.42 | -32.66 |
| 40 | -25.21 | -32.99 | -58.80 | -16.23 | -47.26 | -70.97 | -21.04 | -40.70 | -87.21 | -14.85 | -32.66 | -36.70 | -65.26 | -37.76 |
| 50 | -20.57 | -25.60 | -46.92 | -12.84 | -35.16 | -53.72 | -16.96 | -31.54 | -63.19 | -12.56 | -26.39 | -28.41 | -49.76 | -31.30 |
| 60 | -14.29 | -16.85 | -33.40 | -8.71 | -23.41 | -35.82 | -11.41 | -21.24 | -39.73 | -9.37 | -18.47 | -19.56 | -34.72 | -22.67 |
| 70 | -8.20 | -9.32 | -21.73 | -5.41 | -14.27 | -21.57 | -6.89 | -13.35 | -22.58 | -6.20 | * | -12.21 | -22.62 | -14.52 |

**I_700_ (pA/pF)**

| **Test Potential (mV)** | **cell 1** | **cell 2** | **cell 3** | **cell 4** | **cell 5** | **cell 6** | **cell 7** | **cell 8** | **cell 9** | **cell 10** | **cell 11** | **cell 12** | **cell 13** | **cell 14** |
| --- | --- | --- | --- | --- | --- | --- | --- | --- | --- | --- | --- | --- | --- | --- |
| 0 | -0.153 | -0.103 | -0.147 | -0.215 | -0.318 | -0.174 | -0.150 | -0.179 | -0.192 | -0.116 | -0.145 | -0.120 | -0.265 | -0.082 |
| 10 | -0.311 | -0.263 | -0.504 | -0.618 | -0.996 | -0.628 | -0.250 | -0.837 | -0.607 | -0.238 | -0.730 | -0.499 | -0.682 | -0.207 |
| 20 | -0.983 | -1.798 | -1.665 | -1.342 | -2.872 | -2.131 | -0.744 | -2.421 | -2.211 | -0.624 | -1.981 | -1.589 | -2.757 | -0.874 |
| 30 | -1.627 | -3.450 | -3.782 | -1.759 | -4.462 | -4.794 | -1.605 | -3.461 | -7.371 | -1.024 | -2.897 | -3.106 | -5.866 | -1.817 |
| 40 | -2.292 | -4.185 | -6.323 | -2.194 | -6.016 | -8.233 | -2.156 | -3.963 | -13.023 | -1.279 | -3.347 | -4.334 | -10.288 | -3.071 |
| 50 | -2.652 | -4.694 | -7.887 | -2.479 | -5.921 | -10.074 | -2.796 | -4.624 | -13.879 | -1.492 | -3.379 | -4.851 | -11.447 | -4.002 |
| 60 | -2.337 | -2.767 | -7.507 | -1.990 | -4.858 | -8.297 | -2.489 | -4.092 | -9.876 | -1.478 | -2.898 | -4.137 | -9.539 | -3.833 |
| 70 | -0.782 | * | -5.729 | -1.334 | -3.401 | -5.653 | -1.760 | -2.975 | -5.624 | -1.099 | * | -2.948 | -6.800 | -2.666 |

* cell died

**Fig 3C Ca_V_2.2 with JPH3**

**I_Peak_ (pA/pF)**

| **Test Potential (mV)** | **cell 1** | **cell 2** | **cell 3** | **cell 4** | **cell 5** | **cell 6** | **cell 7** | **cell 8** | **cell 9** | **cell 10** |
| --- | --- | --- | --- | --- | --- | --- | --- | --- | --- | --- |
| -10 | -0.42 | -0.32 | -0.24 | -0.68 | -0.47 | -0.51 | -0.60 | -0.49 | -0.53 | -0.47 |
| 0 | -0.43 | -0.38 | -0.29 | -1.45 | -0.98 | -0.86 | -1.38 | -1.27 | -0.54 | -1.11 |
| 10 | -0.80 | -0.64 | -0.50 | -7.63 | -3.95 | -4.45 | -7.39 | -6.52 | -3.00 | -6.32 |
| 20 | -2.35 | -1.90 | -1.20 | -35.93 | -20.74 | -19.85 | -49.30 | -73.57 | -10.93 | -40.75 |
| 30 | -4.88 | -4.39 | -2.81 | -49.40 | -45.74 | -42.51 | -73.66 | -99.01 | -22.60 | -111.44 |
| 40 | -4.96 | -4.66 | -3.25 | -40.26 | -41.72 | -42.69 | -58.29 | -75.62 | -21.72 | -88.08 |
| 50 | -4.09 | -3.77 | -2.83 | -28.88 | -29.75 | -32.46 | -41.10 | -54.36 | -16.60 | -44.01 |
| 60 | -2.71 | -2.87 | -2.05 | -19.28 | -19.33 | -22.16 | -27.10 | -35.39 | -11.23 | -26.00 |
| 70 | -1.50 | -1.70 | -1.28 | -11.22 | -11.58 | -13.93 | -15.50 | -20.41 | -6.78 | -16.18 |

**I_700_ (pA/pF)**

| **Test Potential (mV)** | **cell 1** | **cell 2** | **cell 3** | **cell 4** | **cell 5** | **cell 6** | **cell 7** | **cell 8** | **cell 9** | **cell 10** |
| --- | --- | --- | --- | --- | --- | --- | --- | --- | --- | --- |
| 0 | -0.168 | -0.099 | -0.126 | -0.301 | -0.248 | -0.269 | -0.362 | -0.220 | -0.223 | -0.524 |
| 10 | -0.249 | -0.204 | -0.232 | -1.936 | -0.574 | -1.123 | -1.603 | -1.341 | -1.276 | -2.640 |
| 20 | -0.574 | -0.510 | -0.536 | -5.874 | -2.199 | -3.557 | -7.074 | -5.933 | -4.258 | -12.689 |
| 30 | -1.062 | -1.236 | -1.028 | -7.899 | -4.453 | -5.848 | -11.749 | -12.195 | -6.969 | -24.378 |
| 40 | -1.145 | -1.451 | -1.359 | -7.509 | -5.475 | -6.621 | -11.244 | -13.083 | -6.600 | -17.273 |
| 50 | -1.138 | -1.258 | -1.287 | -5.608 | -6.131 | -6.128 | -9.624 | -11.205 | -5.227 | -9.764 |
| 60 | -0.932 | -0.856 | -1.095 | -3.532 | -5.413 | -4.883 | -7.176 | -7.646 | -3.755 | -5.650 |
| 70 | -0.595 | -0.740 | -0.719 | -1.366 | -3.872 | -3.361 | -4.228 | -3.486 | -2.250 | -4.176 |

**Fig 3C Ca_V_2.2 with JPH4**

**I_Peak_ (pA/pF)**

| **Test Potential (mV)** | **cell 1** | **cell 2** | **cell 3** | **cell 4** | **cell 5** | **cell 6** | **cell 7** | **cell 8** | **cell 9** | **cell 10** | **cell 11** | **cell 12** |
| --- | --- | --- | --- | --- | --- | --- | --- | --- | --- | --- | --- | --- |
| -10 | -0.65 | -0.65 | -0.60 | -0.43 | -0.94 | -0.54 | -0.79 | -2.27 | -0.88 | -0.35 | -0.65 | -0.64 |
| 0 | -0.60 | -1.14 | -2.53 | -1.41 | -2.26 | -0.78 | -2.36 | -3.16 | -1.94 | -1.01 | -2.11 | -1.44 |
| 10 | -2.07 | -4.41 | -11.75 | -7.74 | -11.91 | -4.29 | -10.17 | -17.83 | -9.76 | -3.72 | -10.10 | -7.70 |
| 20 | -4.74 | -9.86 | -34.44 | -29.66 | -34.92 | -19.77 | -26.05 | -66.54 | -47.79 | -12.83 | -55.01 | -38.22 |
| 30 | -5.98 | -11.22 | -43.14 | -40.45 | -44.74 | -34.46 | -32.19 | -76.23 | -71.34 | -25.38 | -103.62 | -65.44 |
| 40 | -4.91 | -8.95 | -34.66 | -31.93 | -36.24 | -30.43 | -27.02 | -48.95 | -54.42 | -25.30 | -76.90 | -55.88 |
| 50 | -3.64 | -6.38 | -24.32 | -21.63 | -24.32 | -22.32 | -19.05 | -28.48 | -34.26 | -19.65 | -45.24 | -39.24 |
| 60 | -2.55 | -3.89 | -15.08 | -13.54 | -15.96 | -15.12 | -12.35 | -17.09 | -20.10 | -13.50 | -27.40 | -24.86 |
| 70 | -1.39 | -2.23 | -8.21 | -7.76 | -9.67 | -9.21 | -7.14 | -11.62 | -11.72 | -9.02 | -16.27 | -14.47 |

**I_700_ (pA/pF)**

| **Test Potential (mV)** | **cell 1** | **cell 2** | **cell 3** | **cell 4** | **cell 5** | **cell 6** | **cell 7** | **cell 8** | **cell 9** | **cell 10** | **cell 11** | **cell 12** |
| --- | --- | --- | --- | --- | --- | --- | --- | --- | --- | --- | --- | --- |
| 0 | -0.458 | -0.862 | -1.969 | -1.024 | -1.824 | -0.429 | -1.728 | -2.350 | -1.319 | -0.510 | -1.647 | -0.901 |
| 10 | -1.546 | -3.628 | -10.364 | -6.620 | -10.093 | -2.555 | -7.636 | -15.081 | -7.463 | -1.603 | -8.651 | -5.188 |
| 20 | -3.272 | -7.998 | -25.490 | -21.824 | -24.515 | -10.074 | -16.779 | -36.576 | -30.788 | -4.412 | -41.051 | -20.158 |
| 30 | -4.187 | -8.456 | -24.852 | -23.124 | -24.558 | -13.039 | -17.657 | -28.458 | -30.994 | -5.572 | -40.608 | -28.447 |
| 40 | -3.424 | -6.635 | -18.765 | -17.136 | -17.866 | -10.980 | -13.917 | -16.487 | -22.756 | -5.227 | -24.990 | -24.971 |
| 50 | -2.706 | -4.696 | -12.716 | -12.118 | -12.271 | -8.665 | -10.627 | -8.685 | -14.951 | -4.633 | -14.268 | -18.530 |
| 60 | -1.937 | -2.850 | -7.887 | -7.681 | -7.346 | -5.731 | -6.901 | -4.813 | -8.401 | -3.662 | -8.794 | -11.726 |
| 70 | -1.019 | -1.590 | -2.159 | -4.159 | -4.146 | -2.974 | -3.984 | -2.442 | -4.295 | -2.482 | -6.030 | -4.061 |
