# Supplementary material for: Neuronal junctophilins recruit specific CaV and RyR isoforms to ER-PM junctions and functionally alter CaV2.1 and CaV2.2
Source: eLife. 2021 Mar 26;10:e64249. doi: 10.7554/eLife.64249 (PMC8046434; doi:10.7554/eLife.64249)
Supplement: Figure 3—figure supplement 1—source data 1. [file elife-64249-fig3-figsupp1-data1.docx]

**Figure 3-figure supplement 1**

**I_peak_ and I_700_ with Ba^2+^ as charge carrier. (**Data with Ca^2+^ are reported in “Figure 3-source data 1”)

**Ca_V_1.2 without JPHs**

**I_peak_ (pA/pF)**

| **Test Potential** | **cell 1** | **cell 2** | **cell 3** | **cell 4** | **cell 5** | **cell 6** | **cell 7** | **cell 8** | **cell 9** | **cell 10** | **cell 11** | **cell 12** |
| --- | --- | --- | --- | --- | --- | --- | --- | --- | --- | --- | --- | --- |
| 0 | -10.155 | -8.389 | -0.829 | -1.548 | -40.739 | -22.438 | -8.614 | -4.302 | -39.438 | -4.721 | -25.848 | -34.481 |
| 10 | -17.402 | -12.844 | -1.660 | -2.637 | -66.412 | -41.197 | -16.322 | -7.614 | -58.916 | -10.446 | -56.808 | -65.958 |
| 20 | -19.779 | -13.467 | -2.542 | -3.288 | -59.280 | -45.078 | -20.824 | -10.392 | -53.013 | -15.710 | -68.758 | -72.988 |
| 30 | -15.902 | -10.851 | -3.260 | -2.977 | -40.071 | -35.934 | -18.228 | -10.465 | -39.981 | -15.763 | -57.464 | -58.917 |
| 40 | -9.940 | -7.196 | -3.324 | -1.902 | -22.711 | -22.425 | -12.477 | -7.451 | -25.946 | -12.163 | -39.242 | -39.268 |
| 50 | -5.214 | -3.831 | -2.994 | -0.623 | -10.599 | -11.908 | -6.715 | -4.168 | -14.075 | -7.370 | -22.316 | -21.618 |
| 60 | -2.359 | -2.518 | -2.179 | -0.386 | -4.692 | -5.720 | -2.832 | -2.210 | -5.839 | -3.530 | -10.589 | -9.410 |
| 70 | * | * | -1.421 | -0.351 | -1.977 | -3.230 | * | -0.972 | * | -0.729 | -3.502 | -2.603 |

* Cell died

**I_700_ (pA/pF)**

| **Test Potential** | **cell 1** | **cell 2** | **cell 3** | **cell 4** | **cell 5** | **cell 6** | **cell 7** | **cell 8** | **cell 9** | **cell 10** | **cell 11** | **cell 12** |
| --- | --- | --- | --- | --- | --- | --- | --- | --- | --- | --- | --- | --- |
| 0 | -5.707 | -5.582 | -0.435 | -0.892 | -17.586 | -12.767 | -5.204 | -2.624 | -14.103 | -3.379 | -14.550 | -15.129 |
| 10 | -6.510 | -6.253 | -0.711 | -1.107 | -13.251 | -13.691 | -6.829 | -4.268 | -12.565 | -5.417 | -17.285 | -13.019 |
| 20 | -5.655 | -5.739 | -0.786 | -1.083 | -8.144 | -9.796 | -5.777 | -4.318 | -10.159 | -5.547 | -12.979 | -8.759 |
| 30 | -3.922 | -4.032 | -0.797 | -0.763 | -4.679 | -5.660 | -4.050 | -3.514 | -7.439 | -4.629 | -8.339 | -5.846 |
| 40 | -2.569 | -2.569 | -0.794 | -0.345 | -2.199 | -2.700 | -2.352 | -2.303 | -5.041 | -3.168 | -5.145 | -3.475 |
| 50 | -1.086 | -0.340 | -0.891 | -0.059 | -0.441 | -0.547 | -1.139 | -1.095 | -1.956 | -1.677 | -2.939 | -1.941 |
| 60 | -0.518 | 0.000 | -0.728 | -0.069 | -0.273 | -0.225 | -0.432 | -0.564 | -0.329 | -0.558 | -1.412 | -0.994 |
| 70 | * | * | -0.453 | 0.003 | -0.312 | -0.487 | * | -0.158 | * | -0.093 | -0.315 | -0.358 |

* Cell died

**Ca_V_1.2 with JPH4**

**I_peak_ (pA/pF)**

| **Test Potential** | **cell 1** | **cell 2** | **cell 3** | **cell 4** | **cell 5** | **cell 6** | **cell 7** | **cell 8** | **cell 9** | **cell 10** | **cell 11** | **cell 12** |
| --- | --- | --- | --- | --- | --- | --- | --- | --- | --- | --- | --- | --- |
| 0 | -31.659 | -1.896 | -1.368 | -34.462 | -14.089 | -2.814 | -1.352 | -19.219 | -7.995 | -5.632 | -35.981 | -36.702 |
| 10 | -70.067 | -3.515 | -2.651 | -65.614 | -27.683 | -4.766 | -2.455 | -40.345 | -17.490 | -10.913 | -76.377 | -55.306 |
| 20 | -81.776 | -5.021 | -4.462 | -76.019 | -35.931 | -5.621 | -4.078 | -52.640 | -24.493 | -15.216 | -71.594 | -56.450 |
| 30 | -67.901 | -5.343 | -5.326 | -62.952 | -32.773 | -5.173 | -4.962 | -47.073 | -24.647 | -15.857 | -54.186 | -45.028 |
| 40 | -47.430 | -4.367 | -5.048 | -41.627 | -23.358 | -3.564 | -4.406 | -33.368 | -18.814 | -12.710 | -35.938 | -30.454 |
| 50 | -25.969 | -2.979 | -3.773 | -22.901 | -13.774 | -1.764 | -3.391 | -18.983 | -11.265 | -8.449 | -20.324 | -17.536 |
| 60 | -8.491 | 1.753 | -2.071 | -9.135 | -6.009 | -0.646 | -2.123 | -7.867 | -5.589 | -4.414 | -9.434 | -8.261 |
| 70 | * | 1.783 | -0.627 | -1.033 | -2.643 | * | -0.583 | -0.937 | -1.313 | -1.423 | -2.357 | -2.746 |

* Cell died

**I_700_ (pA/pF)**

| **Test Potential** | **cell 1** | **cell 2** | **cell 3** | **cell 4** | **cell 5** | **cell 6** | **cell 7** | **cell 8** | **cell 9** | **cell 10** | **cell 11** | **cell 12** |
| --- | --- | --- | --- | --- | --- | --- | --- | --- | --- | --- | --- | --- |
| 0 | -20.385 | -1.310 | -0.800 | -21.868 | -11.098 | -1.788 | -0.872 | -12.459 | -6.491 | -4.772 | -13.671 | -29.200 |
| 10 | -25.035 | -2.260 | -1.677 | -26.734 | -17.737 | -2.756 | -1.605 | -17.637 | -11.516 | -8.935 | -11.090 | -35.434 |
| 20 | -18.668 | -2.618 | -2.495 | -20.685 | -17.233 | -2.558 | -2.341 | -15.168 | -12.492 | -10.611 | -7.426 | -31.843 |
| 30 | -13.530 | -2.487 | -2.537 | -13.636 | -11.893 | -1.898 | -2.241 | -10.429 | -9.938 | -9.724 | -4.792 | -23.730 |
| 40 | -8.988 | -1.892 | -2.305 | -8.088 | -7.559 | -1.102 | -1.804 | -6.330 | -6.835 | -7.166 | -3.029 | -15.721 |
| 50 | -3.941 | -1.116 | -1.710 | -4.149 | -3.807 | -0.404 | -1.140 | -3.405 | -3.750 | -4.494 | -1.725 | -8.943 |
| 60 | -0.630 | -0.438 | -0.863 | -1.395 | -1.478 | -0.047 | -0.633 | -1.069 | -1.774 | -2.320 | -0.854 | -4.260 |
| 70 | * | -0.127 | -0.143 | -0.073 | -0.749 | * | -0.116 | -0.145 | -0.527 | -0.778 | -0.192 | -1.097 |

* Cell died

**Ca_V_2.1 without JPHs**

**I_peak_ (pA/pF)**

| **Test Potential** | **cell 1** | **cell 2** | **cell 3** | **cell 4** | **cell 5** | **cell 6** | **cell 7** |
| --- | --- | --- | --- | --- | --- | --- | --- |
| 0 | -0.820 | -4.099 | -6.209 | -33.067 | -3.994 | -15.187 | -8.324 |
| 10 | -2.681 | -18.027 | -28.228 | -69.207 | -15.717 | -52.493 | -24.149 |
| 20 | -6.084 | -32.383 | -41.739 | -60.126 | -25.798 | -61.003 | -31.201 |
| 30 | -6.529 | -28.112 | -33.884 | -43.193 | -22.323 | -48.204 | -25.204 |
| 40 | -4.976 | -19.056 | -21.882 | -27.957 | -14.337 | -32.678 | -17.487 |
| 50 | -2.866 | -10.837 | -11.922 | -15.735 | -8.333 | -19.723 | -10.045 |
| 60 | -1.599 | -5.519 | -6.091 | -7.315 | -4.116 | -10.211 | -5.086 |
| 70 | * | -2.273 | -2.547 | -2.287 | -1.657 | -4.442 | -2.358 |

* Cell died

**I_700_ (pA/pF)**

| **Test Potential** | **cell 1** | **cell 2** | **cell 3** | **cell 4** | **cell 5** | **cell 6** | **cell 7** |
| --- | --- | --- | --- | --- | --- | --- | --- |
| 0 | -0.223 | -1.049 | -1.432 | -9.844 | -0.957 | -2.571 | -1.033 |
| 10 | -0.850 | -2.239 | -3.515 | -13.752 | -2.068 | -4.388 | -1.123 |
| 20 | -1.144 | -2.313 | -3.417 | -8.987 | -1.856 | -3.762 | -1.062 |
| 30 | -0.662 | -1.800 | -2.331 | -4.903 | -1.329 | -2.345 | -0.770 |
| 40 | -0.355 | -1.110 | -1.114 | -2.783 | -0.902 | -1.510 | -0.449 |
| 50 | -0.066 | -0.729 | -0.173 | -1.376 | -0.506 | -1.070 | -0.514 |
| 60 | -0.016 | -0.244 | -0.087 | -0.410 | -0.204 | -0.788 | -0.258 |
| 70 | * | -0.168 | -0.116 | -0.196 | -0.046 | -0.450 | -0.112 |

* Cell died

**Ca_V_2.1 with JPH4**

**I_peak_ (pA/pF)**

| **Test Potential** | **cell 1** | **cell 2** | **cell 3** | **cell 4** | **cell 5** | **cell 6** | **cell 7** | **cell 8** | **cell 9** |
| --- | --- | --- | --- | --- | --- | --- | --- | --- | --- |
| 0 | -2.952 | -9.439 | -27.316 | -0.707 | -2.754 | -9.526 | -2.012 | -7.780 | -5.290 |
| 10 | -10.911 | -40.981 | -79.000 | -3.281 | -8.509 | -32.884 | -10.610 | -23.356 | -19.213 |
| 20 | -19.215 | -49.056 | -78.651 | -8.405 | -12.171 | -44.530 | -24.544 | -28.661 | -26.864 |
| 30 | -17.238 | -35.741 | -55.113 | -10.163 | -9.677 | -35.954 | -22.806 | -22.766 | -22.141 |
| 40 | -11.121 | -22.252 | -32.979 | -7.546 | -6.459 | -22.421 | -14.931 | -14.195 | -13.491 |
| 50 | -6.223 | -11.610 | -17.752 | -4.382 | -3.532 | -12.289 | -7.927 | -8.059 | -7.115 |
| 60 | -2.999 | -3.904 | -8.286 | -2.499 | -1.634 | -6.173 | -3.090 | -3.196 | -2.178 |
| 70 | -1.642 | -0.777 | -2.945 | -1.193 | -1.115 | -2.336 | -0.820 | -0.928 | -1.152 |

**I_700_ (pA/pF)**

| **Test Potential** | **cell 1** | **cell 2** | **cell 3** | **cell 4** | **cell 5** | **cell 6** | **cell 7** | **cell 8** | **cell 9** |
| --- | --- | --- | --- | --- | --- | --- | --- | --- | --- |
| 0 | -1.725 | -8.700 | -20.093 | -0.585 | -2.323 | -7.978 | -1.751 | -6.002 | -4.070 |
| 10 | -8.319 | -31.252 | -41.599 | -2.774 | -6.878 | -26.114 | -8.888 | -18.105 | -14.235 |
| 20 | -13.515 | -28.289 | -32.760 | -6.822 | -7.471 | -28.900 | -17.317 | -16.473 | -15.751 |
| 30 | -10.217 | -18.215 | -20.945 | -6.794 | -5.186 | -19.220 | -14.045 | -11.086 | -10.086 |
| 40 | -6.052 | -11.050 | -12.306 | -4.541 | -2.971 | -11.296 | -8.023 | -6.798 | -5.538 |
| 50 | -3.029 | -5.558 | -7.019 | -2.432 | -1.588 | -6.113 | -4.019 | -3.309 | -2.289 |
| 60 | -1.584 | -1.491 | -3.525 | -1.521 | -0.880 | -3.407 | -1.226 | -1.371 | -0.146 |
| 70 | -0.716 | -0.153 | -1.276 | -0.546 | -0.482 | -1.366 | -0.232 | -0.127 | -0.056 |

**Ca_V_2.2 without JPHs**

**I_peak_ (pA/pF)**

| **Test Potential** | **cell 1** | **cell 2** | **cell 3** | **cell 4** | **cell 5** | **cell 6** | **cell 7** |
| --- | --- | --- | --- | --- | --- | --- | --- |
| 0 | -1.141 | -2.770 | -2.952 | -5.522 | -14.580 | -5.356 | -4.173 |
| 10 | -3.919 | -9.739 | -21.878 | -23.396 | -101.995 | -29.527 | -21.392 |
| 20 | -9.676 | -25.059 | -33.427 | -35.564 | -114.982 | -55.893 | -38.019 |
| 30 | -13.528 | -30.518 | -28.975 | -30.060 | -92.015 | -46.745 | -33.442 |
| 40 | -12.865 | -24.564 | -21.877 | -20.903 | -63.939 | -34.041 | -25.001 |
| 50 | -9.736 | -16.629 | -13.885 | -13.333 | -41.463 | -21.989 | -16.381 |
| 60 | -6.078 | -9.143 | -7.268 | -6.841 | -23.513 | -12.846 | -9.178 |
| 70 | -3.481 | * | -2.092 | -2.942 | -12.040 | -6.846 | -3.809 |

* Cell died

**I_700_ (pA/pF)**

| **Test Potential** | **cell 1** | **cell 2** | **cell 3** | **cell 4** | **cell 5** | **cell 6** | **cell 7** |
| --- | --- | --- | --- | --- | --- | --- | --- |
| 0 | -0.345 | -0.615 | -1.393 | -3.742 | -3.779 | -2.046 | -0.637 |
| 10 | -1.006 | -2.117 | -6.336 | -15.362 | -13.550 | -10.421 | -3.125 |
| 20 | -1.531 | -3.353 | -9.942 | -16.846 | -14.906 | -15.257 | -5.071 |
| 30 | -2.057 | -3.705 | -7.633 | -9.833 | -10.100 | -11.030 | -4.440 |
| 40 | -2.095 | -2.846 | -4.795 | -5.146 | -5.778 | -6.828 | -2.844 |
| 50 | -1.755 | -1.978 | -2.431 | -2.458 | -3.384 | -4.213 | -1.805 |
| 60 | -1.124 | -0.747 | -0.660 | -1.083 | -1.654 | -1.994 | -0.978 |
| 70 | -0.298 | * | -0.129 | -0.357 | -0.672 | -0.472 | -0.312 |

* Cell died

**Ca_V_2.2 with JPH4**

**I_peak_ (pA/pF)**

| **Test Potential** | **cell 1** | **cell 2** | **cell 3** | **cell 4** | **cell 5** | **cell 6** | **cell 7** | **cell 8** | **cell 9** |
| --- | --- | --- | --- | --- | --- | --- | --- | --- | --- |
| 0 | -1.236 | -0.773 | -6.670 | -7.559 | -16.345 | -4.078 | -9.758 | -3.380 | -2.608 |
| 10 | -7.081 | -5.915 | -31.987 | -53.131 | -68.335 | -17.137 | -48.087 | -14.054 | -8.029 |
| 20 | -54.049 | -20.280 | -61.978 | -55.590 | -59.860 | -33.605 | -81.514 | -23.308 | -15.636 |
| 30 | -93.309 | -23.975 | -56.084 | -40.027 | -42.774 | -30.446 | -70.296 | -23.149 | -17.692 |
| 40 | -72.993 | -19.272 | -38.705 | -26.622 | -26.198 | -22.264 | -49.756 | -18.520 | -13.885 |
| 50 | -47.834 | -12.378 | -22.705 | -15.991 | -14.963 | -14.365 | -30.341 | -13.517 | -9.938 |
| 60 | -26.818 | -5.745 | -11.194 | -7.981 | -7.160 | -8.031 | -16.334 | -7.967 | -5.605 |
| 70 | -11.397 | * | -5.534 | -2.809 | * | -3.837 | -6.716 | -3.902 | -3.093 |

* Cell died

**I_700_ (pA/pF)**

| **Test Potential** | **cell 1** | **cell 2** | **cell 3** | **cell 4** | **cell 5** | **cell 6** | **cell 7** | **cell 8** | **cell 9** |
| --- | --- | --- | --- | --- | --- | --- | --- | --- | --- |
| 0 | -0.785 | -0.273 | -5.403 | -6.272 | -15.235 | -2.492 | -8.932 | -2.708 | -1.476 |
| 10 | -5.418 | -5.163 | -28.125 | -40.243 | -53.734 | -10.520 | -44.850 | -12.581 | -5.088 |
| 20 | -40.554 | -17.578 | -43.487 | -34.847 | -41.530 | -15.588 | -52.884 | -16.545 | -7.529 |
| 30 | -60.250 | -19.205 | -35.210 | -23.088 | -26.737 | -12.259 | -36.830 | -12.815 | -6.709 |
| 40 | -44.085 | -13.470 | -23.117 | -13.905 | -15.233 | -7.578 | -21.939 | -7.677 | -4.136 |
| 50 | -26.966 | -7.524 | -13.131 | -7.762 | -7.686 | -4.338 | -12.654 | -4.512 | -2.966 |
| 60 | -14.843 | -2.160 | -6.190 | -3.332 | -2.427 | -2.283 | -5.903 | -2.534 | -1.685 |
| 70 | -5.047 | * | -1.821 | -0.640 | * | -1.010 | -1.857 | -1.198 | -0.755 |

* Cell died
