# Supplementary material for: Neuronal junctophilins recruit specific CaV and RyR isoforms to ER-PM junctions and functionally alter CaV2.1 and CaV2.2
Source: eLife. 2021 Mar 26;10:e64249. doi: 10.7554/eLife.64249 (PMC8046434; doi:10.7554/eLife.64249)
Supplement: Figure 4—source data 1. [file elife-64249-fig4-data1.docx]

**Figure 4 Data for Ca_V_2.1 and Ca_V_2.2 with truncated junctophilins.**

(Data for Ca_V_2.1 and Ca_V_2.2 w/o JPHs in “Figure 3-source data 1”).

**Fig 4A Ca_V_2.1 with JPH3(1-707)**

**I_Peak_ (pA/pF)**

| **Test Potential (mV)l** | **cell 1** | **cell 2** | **cell 3** | **cell 4** | **cell 5** | **cell 6** | **cell 7** | **cell 8** | **cell 9** | **cell 10** | **cell 11** | **cell 12** | **cell 13** | **cell 14** |
| --- | --- | --- | --- | --- | --- | --- | --- | --- | --- | --- | --- | --- | --- | --- |
| -10 | -0.78 | -0.58 | -0.08 | -0.96 | -0.44 | -0.43 | -0.53 | -0.43 | -0.36 | -0.32 | -0.20 | -0.40 | -0.36 | -0.59 |
| 0 | -0.86 | -1.32 | -0.18 | -0.56 | -0.72 | -0.49 | -1.10 | -1.39 | -0.46 | -0.89 | -0.45 | -1.10 | -1.11 | -1.27 |
| 10 | -3.97 | -6.83 | -0.47 | -3.44 | -2.04 | -2.93 | -3.95 | -7.08 | -1.79 | -4.86 | -2.13 | -4.03 | -5.46 | -8.75 |
| 20 | -15.69 | -26.00 | -1.36 | -6.82 | -3.97 | -12.50 | -6.04 | -17.42 | -4.38 | -17.31 | -8.76 | -18.74 | -27.43 | -29.96 |
| 30 | -24.32 | -32.97 | -2.37 | -7.18 | -3.96 | -19.18 | -5.98 | -18.86 | -4.70 | -22.83 | -16.00 | -31.39 | -44.12 | -40.16 |
| 40 | -21.19 | -26.21 | -1.99 | -5.36 | -3.22 | -16.06 | -4.42 | -15.61 | -4.07 | -18.85 | -15.02 | -26.10 | -36.06 | -31.92 |
| 50 | -14.19 | -17.56 | -1.32 | -3.66 | -2.04 | -10.04 | -2.83 | -10.08 | -2.98 | -12.98 | -10.55 | -19.34 | -24.55 | -21.37 |
| 60 | -8.13 | -9.98 | -0.87 | -2.19 | -1.19 | -5.05 | -1.73 | -5.85 | -1.56 | -7.96 | -6.65 | -10.07 | -14.43 | -12.00 |
| 70 | -3.91 | -4.81 | -0.38 | * | -0.79 |  | -0.93 | -2.52 | * | -4.05 | -3.60 | -5.32 | -6.56 | -5.60 |

* cell died

**I_700_ (pA/pF)**

| **Test Potential (mV)** | **cell 1** | **cell 2** | **cell 3** | **cell 4** | **cell 5** | **cell 6** | **cell 7** | **cell 8** | **cell 9** | **cell 10** | **cell 11** | **cell 12** | **cell 13** | **cell 14** |
| --- | --- | --- | --- | --- | --- | --- | --- | --- | --- | --- | --- | --- | --- | --- |
| 0 | -0.566 | -0.934 | -0.125 | -0.473 | -0.399 | -0.303 | -0.795 | -1.162 | -0.373 | -0.629 | -0.338 | -0.460 | -0.792 | -0.851 |
| 10 | -3.057 | -5.647 | -0.235 | -2.817 | -1.739 | -2.176 | -3.714 | -6.364 | -1.428 | -4.225 | -1.542 | -2.585 | -4.180 | -6.159 |
| 20 | -10.201 | -16.771 | -0.875 | -5.713 | -3.022 | -7.702 | -5.137 | -12.856 | -3.432 | -13.742 | -4.608 | -8.455 | -17.164 | -14.972 |
| 30 | -12.738 | -16.808 | -1.172 | -4.460 | -2.264 | -8.106 | -3.232 | -10.089 | -2.775 | -14.301 | -5.499 | -9.936 | -21.749 | -14.226 |
| 40 | -10.104 | -12.250 | -0.877 | -2.375 | -1.258 | -5.855 | -1.770 | -6.493 | -1.713 | -10.049 | -4.906 | -7.973 | -17.618 | -10.533 |
| 50 | -6.416 | -8.006 | -0.572 | -1.117 | -0.709 | -2.984 | -1.015 | -3.707 | -1.162 | -5.336 | -3.663 | -5.289 | -12.143 | -6.772 |
| 60 | -3.480 | -4.478 | -0.327 | -0.457 | -0.311 | -0.510 | -0.780 | -1.829 | -0.386 | -2.152 | -2.458 | -3.164 | -6.979 | -3.464 |
| 70 | -1.124 | -1.859 | -0.198 | * | 0.005 | * | -0.309 | -0.615 | * | * | -1.475 | -1.153 | -2.401 | -1.129 |

* cell died

**Fig 4A Ca_V_2.1 with JPH4(1-576)**

**I_Peak_ (pA/pF)**

| **Test Potential (mV)** | **cell 1** | **cell 2** | **cell 3** | **cell 4** | **cell 5** | **cell 6** | **cell 7** | **cell 8** | **cell 9** | **cell 10** | **cell 11** | **cell 12** | **cell 13** | **cell 14** | **cell 15** | **cell 16** | **cell 17** | **cell 18** |
| --- | --- | --- | --- | --- | --- | --- | --- | --- | --- | --- | --- | --- | --- | --- | --- | --- | --- | --- |
| -10 | -0.70 | -0.15 | -0.33 | -0.64 | -0.41 | -0.43 | -0.50 | -0.23 | -0.62 | -0.35 | -0.39 | -0.36 | -0.43 | -0.42 | -0.40 | -0.54 | -0.91 | -0.55 |
| 0 | -1.41 | -0.31 | -0.87 | -0.92 | -1.09 | -1.28 | -2.34 | -1.53 | -2.85 | -0.19 | -0.43 | -0.54 | -1.58 | -0.89 | -0.50 | -2.10 | -1.13 | -0.61 |
| 10 | -6.75 | -0.89 | -2.64 | -1.43 | -5.74 | -7.92 | -14.62 | -9.04 | -15.59 | -1.84 | -1.10 | -1.22 | -9.88 | -4.32 | -1.56 | -10.41 | -4.55 | -2.22 |
| 20 | -30.90 | -2.65 | -9.59 | -3.89 | -18.03 | -27.39 | -38.22 | -24.26 | -40.15 | -6.64 | -3.17 | -3.21 | -33.54 | -13.30 | -4.27 | -23.22 | -17.82 | -7.83 |
| 30 | -49.61 | -3.91 | -15.17 | -5.32 | -23.51 | -35.37 | -39.18 | -28.78 | -43.52 | -10.05 | -5.00 | -4.65 | -37.37 | -16.64 | -5.60 | -24.59 | -24.89 | -13.24 |
| 40 | -41.44 | -3.35 | -13.57 | -4.42 | -18.64 | -28.14 | -28.91 | -21.39 | -32.73 | -8.21 | -4.29 | -3.99 | -27.47 | -13.78 | -4.23 | -18.48 | -20.12 | -11.94 |
| 50 | -27.45 | -2.38 | -9.47 | -3.07 | -11.94 | -18.25 | -18.08 | -13.72 | -21.25 | -5.32 | -3.04 | -2.78 | -17.51 | -9.12 | -3.15 | -12.12 | -13.15 | -8.24 |
| 60 | -15.66 | -1.32 | -5.52 | -2.08 | -6.48 | -10.36 | -10.36 | -6.81 | -12.38 | -2.59 | -1.76 | -1.81 | -9.75 | -5.25 | -1.90 | -6.90 | -7.82 | -5.01 |
| 70 | -8.16 | -0.81 | -3.39 | -0.94 | -2.74 | -4.86 | -4.35 | -2.73 | -6.28 | * | -0.99 | -0.97 | -4.87 | -2.51 | -1.02 | -3.77 | -4.20 | -2.75 |

* cell died

**I_700_ (pA/pF)**

| **Test Potential (mV)** | **cell 1** | **cell 2** | **cell 3** | **cell 4** | **cell 5** | **cell 6** | **cell 7** | **cell 8** | **cell 9** | **cell 10** | **cell 11** | **cell 12** | **cell 13** | **cell 14** | **cell 15** | **cell 16** | **cell 17** | **cell 18** |
| --- | --- | --- | --- | --- | --- | --- | --- | --- | --- | --- | --- | --- | --- | --- | --- | --- | --- | --- |
| 0 | -0.926 | -0.250 | -0.541 | -0.662 | -0.873 | -1.099 | -2.061 | -1.313 | -2.285 | -0.178 | -0.309 | -0.290 | -1.110 | -0.634 | -0.289 | -1.842 | -0.636 | -0.314 |
| 10 | -5.941 | -0.666 | -2.294 | -1.009 | -5.073 | -7.568 | -14.164 | -6.866 | -14.281 | -1.491 | -0.935 | -0.885 | -9.271 | -3.767 | -1.278 | -9.291 | -4.128 | -1.616 |
| 20 | -25.034 | -2.065 | -8.272 | -3.155 | -14.432 | -25.359 | -27.329 | -13.908 | -26.671 | -5.796 | -2.859 | -2.760 | -26.278 | -11.286 | -3.673 | -16.134 | -16.642 | -6.068 |
| 30 | -29.805 | -2.471 | -11.062 | -3.531 | -14.684 | -26.331 | -21.076 | -12.058 | -21.013 | -6.960 | -3.826 | -3.463 | -22.291 | -10.752 | -3.712 | -12.825 | -18.934 | -8.129 |
| 40 | -21.411 | -1.792 | -8.623 | -2.434 | -10.506 | -18.090 | -13.628 | -8.099 | -14.152 | -4.956 | -2.996 | -2.683 | -14.660 | -6.798 | -2.392 | -8.357 | -13.823 | -6.440 |
| 50 | -13.630 | -1.093 | -5.987 | -1.627 | -6.130 | -11.056 | -7.627 | -4.722 | -8.787 | -2.793 | -1.985 | -1.623 | -8.939 | -4.063 | -1.334 | -5.266 | -8.768 | -4.312 |
| 60 | -7.426 | -0.630 | -3.520 | -1.050 | -3.113 | -5.789 | -3.690 | -2.055 | -4.980 | -0.793 | -1.201 | -1.048 | -4.407 | -1.850 | -0.773 | -2.917 | -5.020 | -2.590 |
| 70 | -3.821 | -0.427 | -2.120 | -0.382 | -1.204 | -2.582 | -0.802 | -0.157 | -1.721 | * | -0.741 | -0.650 | -1.781 | -0.463 | -0.415 | -1.734 | -2.595 | -1.419 |

* cell died

**Fig 4B**

**Ca_V_2.2 with JPH3(1-707)**

**I_Peak_ (pA/pF)**

| **Test Potential (mV)** | **cell 1** | **cell 2** | **cell 3** | **cell 4** | **cell 5** | **cell 6** | **cell 7** | **cell 8** | **cell 9** | **cell 10** | **cell 11** | **cell 12** | **cell 13** | **cell 14** |
| --- | --- | --- | --- | --- | --- | --- | --- | --- | --- | --- | --- | --- | --- | --- |
| -10 | -0.24 | -0.48 | -0.75 | -0.72 | -0.56 | -0.42 | -0.66 | -0.99 | -0.35 | -0.55 | -0.26 | -0.54 | -0.43 | -0.25 |
| 0 | -0.54 | -1.16 | -1.48 | -2.03 | -0.90 | -0.99 | -1.98 | -1.87 | -0.89 | -1.17 | -0.23 | -1.65 | -1.92 | -0.65 |
| 10 | -1.84 | -5.78 | -8.05 | -13.91 | -3.16 | -3.39 | -10.54 | -10.54 | -4.36 | -6.09 | -0.39 | -7.37 | -11.14 | -2.91 |
| 20 | -8.02 | -21.83 | -46.75 | -70.54 | -15.94 | -12.56 | -68.39 | -66.09 | -14.41 | -31.94 | -0.85 | -31.64 | -35.40 | -12.20 |
| 30 | -17.31 | -34.33 | -111.69 | -64.38 | -53.44 | -22.30 | -107.40 | -87.81 | -22.47 | -73.94 | -1.60 | -58.66 | -45.73 | -22.53 |
| 40 | -17.06 | -31.31 | -92.16 | -38.64 | -58.00 | -21.79 | -78.29 | -64.77 | -22.46 | -65.51 | -1.88 | -52.50 | -38.51 | -21.34 |
| 50 | -13.22 | -23.27 | -59.54 | -26.25 | -45.02 | -15.86 | -41.50 | -38.33 | -17.36 | -46.15 | -1.59 | -38.17 | -27.78 | -16.26 |
| 60 | -8.74 | -15.17 | -33.58 | -17.75 | -31.89 | -10.35 | -22.70 | -21.11 | -12.54 | -29.44 | -1.02 | -24.69 | -17.98 | -10.86 |
| 70 | -4.86 | -10.70 | -18.37 | -11.51 | -19.66 | -6.21 | -13.22 | -11.69 | -7.55 | -17.44 | -0.66 | -14.65 | -10.88 | -6.50 |

**I_700_ (pA/pF)**

| **Test Potential (mV)** | **cell 1** | **cell 2** | **cell 3** | **cell 4** | **cell 5** | **cell 6** | **cell 7** | **cell 8** | **cell 9** | **cell 10** | **cell 11** | **cell 12** | **cell 13** | **cell 14** |
| --- | --- | --- | --- | --- | --- | --- | --- | --- | --- | --- | --- | --- | --- | --- |
| 0 | -0.190 | -0.586 | -0.521 | -0.803 | -0.313 | -0.172 | -0.781 | -0.778 | -0.571 | -0.431 | -0.058 | -0.679 | -0.907 | -0.359 |
| 10 | -1.106 | -2.632 | -2.605 | -6.385 | -1.096 | -0.489 | -3.151 | -3.792 | -2.793 | -2.258 | -0.120 | -2.973 | -4.547 | -1.552 |
| 20 | -3.622 | -8.275 | -12.126 | -17.412 | -4.215 | -1.344 | -14.276 | -17.041 | -7.696 | -9.621 | -0.193 | -10.301 | -12.175 | -5.215 |
| 30 | -6.576 | -11.519 | -28.084 | -12.821 | -10.897 | -2.046 | -25.067 | -26.431 | -9.938 | -18.373 | -0.371 | -16.588 | -16.064 | -8.464 |
| 40 | -6.742 | -11.965 | -27.111 | -6.251 | -15.391 | -2.363 | -20.542 | -21.213 | -9.605 | -17.846 | -0.451 | -16.692 | -14.859 | -8.519 |
| 50 | -5.320 | -10.147 | -19.125 | -3.435 | -14.721 | -2.056 | -10.969 | -13.258 | -6.674 | -14.223 | -0.463 | -13.403 | -11.017 | -6.642 |
| 60 | -3.393 | -6.993 | -11.485 | -1.272 | -11.696 | -1.832 | -5.094 | -6.961 | -4.079 | -9.933 | -0.303 | -9.074 | -7.198 | -4.638 |
| 70 | -1.600 | -4.294 | -6.372 | 0.070 | -7.443 | -0.934 | -2.483 | -3.763 | -2.636 | -6.070 | -0.238 | -5.591 | -4.182 | -2.906 |

**Fig 4B Ca_V_2.2 with JPH4(1-576)**

**I_Peak_ (pA/pF)**

| **Test Potential (mV)** | **cell 1** | **cell 2** | **cell 3** | **cell 4** | **cell 5** | **cell 6** | **cell 7** | **cell 8** | **cell 9** | **cell 10** | **cell 11** |
| --- | --- | --- | --- | --- | --- | --- | --- | --- | --- | --- | --- |
| -10 | -0.64 | -0.61 | -0.63 | -0.48 | -0.34 | -0.75 | -0.25 | -0.44 | -0.46 | -0.50 | -0.83 |
| 0 | -1.16 | -1.44 | -1.46 | -1.05 | -0.85 | -1.26 | -0.57 | -1.44 | -0.61 | -0.82 | -1.82 |
| 10 | -5.98 | -7.14 | -7.55 | -5.65 | -4.49 | -7.65 | -3.04 | -8.16 | -3.11 | -4.47 | -11.79 |
| 20 | -26.58 | -30.04 | -33.91 | -23.87 | -16.55 | -39.21 | -12.01 | -39.19 | -12.63 | -23.77 | -52.50 |
| 30 | -51.72 | -58.48 | -60.47 | -39.16 | -28.76 | -59.63 | -21.91 | -72.76 | -27.89 | -45.09 | -67.48 |
| 40 | -44.76 | -51.79 | -51.12 | -33.37 | -25.95 | -45.89 | -19.93 | -60.68 | -28.97 | -39.15 | -49.72 |
| 50 | -31.13 | -36.37 | -33.64 | -23.81 | -19.00 | -29.79 | -14.00 | -41.96 | -21.99 | -27.61 | -31.64 |
| 60 | -19.81 | -22.85 | -19.52 | -15.35 | -12.12 | -17.60 | -8.80 | -26.25 | -14.87 | -17.79 | -19.03 |
| 70 | -11.43 | -13.12 | -10.65 | -9.21 | -6.77 | -10.19 | -3.76 | -15.26 | -8.56 | -10.07 | -11.32 |

**I_700_ (pA/pF)**

| **Test Potential (mV)** | **cell 1** | **cell 2** | **cell 3** | **cell 4** | **cell 5** | **cell 6** | **cell 7** | **cell 8** | **cell 9** | **cell 10** | **cell 11** |
| --- | --- | --- | --- | --- | --- | --- | --- | --- | --- | --- | --- |
| 0 | -0.918 | -1.180 | -0.912 | -0.781 | -0.550 | -1.057 | -0.349 | -0.877 | -0.378 | -0.317 | -1.368 |
| 10 | -4.638 | -5.579 | -5.263 | -4.529 | -3.371 | -6.607 | -2.215 | -5.340 | -2.418 | -2.281 | -9.578 |
| 20 | -20.909 | -23.399 | -23.057 | -17.871 | -11.278 | -32.389 | -9.298 | -23.790 | -8.374 | -10.518 | -27.434 |
| 30 | -29.873 | -34.926 | -30.667 | -23.480 | -14.761 | -35.068 | -13.422 | -33.130 | -15.154 | -16.403 | -22.684 |
| 40 | -23.048 | -27.096 | -23.332 | -18.208 | -11.870 | -23.972 | -10.749 | -26.592 | -13.214 | -14.399 | -15.362 |
| 50 | -15.402 | -17.698 | -14.918 | -12.207 | -7.673 | -14.609 | -6.653 | -18.255 | -10.175 | -10.539 | -10.063 |
| 60 | -9.267 | -10.426 | -8.181 | -7.352 | -3.879 | -8.132 | -3.481 | -10.675 | -6.726 | -6.911 | -6.242 |
| 70 | -4.555 | -4.965 | -4.126 | -3.962 | -1.267 | -4.106 | -0.541 | -5.154 | -4.013 | -3.378 | -3.457 |
