# Supplementary material for: Neuronal junctophilins recruit specific CaV and RyR isoforms to ER-PM junctions and functionally alter CaV2.1 and CaV2.2
Source: eLife. 2021 Mar 26;10:e64249. doi: 10.7554/eLife.64249 (PMC8046434; doi:10.7554/eLife.64249)
Supplement: Figure 5—source data 1. [file elife-64249-fig5-data1.docx]

**Figure 5C RyR1, RyR2 & RyR3 vs JPH3 & JPH4**

| **Cell** | **RyR1 vs** | | **RyR2 vs** | | **RyR3 vs** | |
| --- | --- | --- | --- | --- | --- | --- |
|  | **JPH3** | **JPH4** | **JPH3** | **JPH4** | **JPH3** | **JPH4** |
| 1 | 0.73 | 0.3200 | 0.64 | 0.2400 | 0.77 | 0.72 |
| 2 | 0.91 | 0.3600 | 0.58 | 0.2100 | 0.56 | 0.52 |
| 3 | 0.67 | 0.2500 | 0.45 | 0.0500 | 0.69 | 0.43 |
| 4 | 0.87 | 0.2600 | 0.63 | 0.1600 | 0.72 | 0.46 |
| 5 | 0.85 | 0.3200 | 0.57 | 0.3400 | 0.59 | 0.31 |
| 6 | 0.66 | 0.2900 | 0.59 | 0.2600 | 0.79 | 0.69 |
| 7 | 0.86 | 0.4400 | 0.50 | 0.3700 | 0.86 | 0.69 |
| 8 | 0.91 | 0.4400 | 0.53 | 0.2800 | 0.62 | 0.46 |
| 9 | 0.93 | 0.3000 | 0.44 | 0.1600 | 0.62 | 0.54 |
| 10 | 0.84 | 0.3400 | 0.49 | 0.4400 | 0.72 | 0.40 |
| 11 | 0.90 | 0.1900 | 0.53 | 0.0300 | 0.69 | 0.63 |
| 12 | 0.84 | 0.1100 | 0.44 | 0.3100 | 0.81 | 0.54 |
| 13 | 0.87 | 0.4200 | 0.59 | 0.3700 | 0.70 | 0.44 |
| 14 | 0.91 | 0.4300 | 0.53 | 0.2200 | 0.77 | 0.36 |
| 15 | 0.87 | 0.3000 | 0.31 | 0.3900 | 0.71 | 0.56 |
| 16 | 0.81 | 0.4200 | 0.53 | 0.3100 | 0.65 | 0.58 |
| 17 | 0.81 |  | 0.47 | 0.1900 | 0.67 | 0.57 |
| 18 | 0.89 |  | 0.64 | 0.1400 | 0.81 | 0.49 |
| 19 | 0.85 |  | 0.48 | 0.1100 | 0.80 | 0.64 |
| 20 | 0.64 |  | 0.59 | 0.2800 | 0.66 | 0.60 |
| 21 | 0.85 |  | 0.56 | 0.3600 | 0.77 | 0.42 |
| 22 | 0.81 |  | 0.36 | 0.2200 | 0.64 | 0.56 |
| 23 | 0.26 |  | 0.47 |  | 0.71 | 0.68 |
| 24 | 0.55 |  |  |  | 0.79 | 0.68 |
| 25 | 0.77 |  |  |  | 0.73 | 0.58 |
| 26 | 0.78 |  |  |  | 0.73 | 0.48 |
| 27 |  |  |  |  | 0.57 | 0.56 |
| 28 |  |  |  |  | 0.64 | 0.50 |
| 29 |  |  |  |  | 0.81 |  |
| 30 |  |  |  |  | 0.68 |  |
| 31 |  |  |  |  | 0.59 |  |

**Pearson’s Coefficients**

**Statistics** **One-way ANOVA:** p < 0.0001

| **Tukey's multiple comparisons test** | **Mean Diff.** | **95% CI of diff.** | **Significant?** | **Summary** | **Adjusted p Value** |
| --- | --- | --- | --- | --- | --- |
| [JPH3 vs RyR1] vs [JPH3 vs RyR2] | 0.2756 | 0.1880 to 0.3631 | Yes | **** | < 0.0001 |
| [JPH3 vs RyR1] vs [JPH3 vs RyR3] | 0.08836 | 0.007023 to 0.1697 | Yes | * | 0.0248 |
| [JPH3 vs RyR1] vs [JPH4 vs RyR1] | 0.4695 | 0.3723 to 0.5667 | Yes | **** | < 0.0001 |
| [JPH3 vs RyR1] vs [JPH4 vs RyR2] | 0.5466 | 0.4580 to 0.6352 | Yes | **** | < 0.0001 |
| [JPH3 vs RyR1] vs [JPH4 vs RyR3] | 0.2549 | 0.1716 to 0.3382 | Yes | **** | < 0.0001 |
| [JPH3 vs RyR2] vs [JPH3 vs RyR3] | -0.1872 | -0.2714 to -0.1030 | Yes | **** | < 0.0001 |
| [JPH3 vs RyR2] vs [JPH4 vs RyR1] | 0.1939 | 0.09431 to 0.2935 | Yes | **** | < 0.0001 |
| [JPH3 vs RyR2] vs [JPH4 vs RyR2] | 0.2710 | 0.1798 to 0.3622 | Yes | **** | < 0.0001 |
| [JPH3 vs RyR2] vs [JPH4 vs RyR3] | -0.02067 | -0.1067 to 0.06541 | No | ns | 0.9824 |
| [JPH3 vs RyR3] vs [JPH4 vs RyR1] | 0.3811 | 0.2870 to 0.4753 | Yes | **** | < 0.0001 |
| [JPH3 vs RyR3] vs [JPH4 vs RyR2] | 0.4582 | 0.3729 to 0.5435 | Yes | **** | < 0.0001 |
| [JPH3 vs RyR3] vs [JPH4 vs RyR3] | 0.1666 | 0.08681 to 0.2463 | Yes | **** | < 0.0001 |
| [JPH4 vs RyR1] vs [JPH4 vs RyR2] | 0.07710 | -0.02339 to 0.1776 | No | ns | 0.2366 |
| [JPH4 vs RyR1] vs [JPH4 vs RyR3] | -0.2146 | -0.3104 to -0.1187 | Yes | **** | < 0.0001 |
| [JPH4 vs RyR2] vs [JPH4 vs RyR3] | -0.2917 | -0.3788 to -0.2045 | Yes | **** | < 0.0001 |
