# Supplementary material for: Neuronal junctophilins recruit specific CaV and RyR isoforms to ER-PM junctions and functionally alter CaV2.1 and CaV2.2
Source: eLife. 2021 Mar 26;10:e64249. doi: 10.7554/eLife.64249 (PMC8046434; doi:10.7554/eLife.64249)
Supplement: Figure 5—figure supplement 1—source data 1. [file elife-64249-fig5-figsupp1-data1.docx]

**Figure 5-figure supplement 1.**

ΔF/F_0_ vs. time

|  | tsA201 without stimulation | | | | | | | | | | tsA201 +caffeine | | | | | | | | | | RyR1-stable + caffeine | | | | | | | | | |
| --- | --- | --- | --- | --- | --- | --- | --- | --- | --- | --- | --- | --- | --- | --- | --- | --- | --- | --- | --- | --- | --- | --- | --- | --- | --- | --- | --- | --- | --- | --- |
| **sec** | **Cell1** | **Cell2** | **Cell3** | **Cell4** | **Cell5** | **Cell6** | **Cell7** | **Cell8** | **Cell9** | **Cell10** | **Cell1** | **Cell2** | **Cell3** | **Cell4** | **Cell5** | **Cell6** | **Cell7** | **Cell8** | **Cell9** | **Cell10** | **Cell1** | **Cell2** | **Cell3** | **Cell4** | **Cell5** | **Cell6** | **Cell7** | **Cell8** | **Cell9** | **Cell10** |
| 0.00 | -0.00161 | -0.00046 | 0.010924 | -0.00046 | 0.004082 | 0.002451 | -0.00466 | -0.00705 | -0.01700 | -0.00693 | -0.00145 | -0.00759 | -0.00511 | -0.00973 | -0.00759 | 0.005189 | -0.01156 | -0.00149 | -0.00318 | -0.00236 | -0.00207 | -0.02920 | 0.023458 | -0.01048 | -0.03758 | -0.01904 | 0.042583 | -0.01177 | -0.01892 | 0.007258 |
| 0.25 | -0.00634 | -0.01561 | 0.00920 | 0.002591 | -0.00030 | 0.010423 | -0.00011 | -0.01231 | -0.01229 | -0.00890 | -0.00391 | -0.00442 | -0.00650 | -0.01227 | -0.00442 | -0.00256 | -0.01442 | -0.01164 | 0.0000535 | -0.00138 | -0.00995 | -0.02135 | 0.02436 | -0.01306 | -0.02582 | -0.00849 | 0.027032 | -0.02042 | -0.01867 | 0.008004 |
| 0.50 | -0.00341 | 0.000282 | 0.004143 | -0.01054 | -0.00733 | 0.002515 | 0.00071 | -0.00999 | -0.00285 | -0.00824 | 0.000495 | -0.00538 | -0.01028 | -0.00885 | -0.00538 | 0.004477 | -0.01261 | -0.00575 | -0.00642 | -0.00338 | -0.02195 | -0.01649 | 0.004301 | -0.00770 | -0.02245 | -0.02154 | -0.00143 | 0.002993 | -0.00262 | 0.00069 |
| 0.75 | -0.00679 | -0.00713 | -0.00134 | 0.00011 | -0.00061 | 0.008751 | -0.01162 | -0.00252 | -0.00427 | -0.00206 | -0.00547 | -0.00689 | 0.003075 | -0.00659 | -0.00689 | -0.00063 | -0.00134 | -0.00334 | -0.00694 | 0.004189 | -0.00420 | -0.01245 | -0.00193 | -0.00031 | -0.03053 | -0.00192 | -0.01170 | -0.01037 | -0.00161 | -0.00553 |
| 1.00 | 0.003226 | 0.005361 | -0.00976 | 0.007604 | 0.011957 | -0.00501 | -0.00461 | 0.000516 | -0.00234 | -0.00299 | -0.00035 | 0.004432 | -0.00251 | 0.004301 | 0.004432 | -0.00373 | -0.00067 | -0.00107 | -0.00128 | -0.00146 | -0.00152 | -0.01975 | -0.00231 | -0.00266 | -0.02245 | 0.007598 | -0.01527 | 0.008678 | 0.015125 | 0.003078 |
| 1.25 | 0.004998 | 0.00319 | -0.00976 | -0.00325 | -0.00052 | -0.00224 | 0.006645 | 0.002632 | 0.002961 | 0.003236 | -0.00083 | 0.00723 | 0.003823 | 0.000776 | 0.00723 | -0.00160 | 0.003783 | 0.000411 | 0.005955 | 0.005212 | -0.00196 | 0.010753 | -0.00515 | 0.010277 | -0.01912 | 0.019668 | -0.01307 | 0.012543 | 0.013728 | 0.006611 |
| 1.50 | 0.00474 | 0.010522 | -0.00490 | -0.00227 | 0.000807 | -0.01137 | 0.003575 | 0.016977 | 0.019163 | 0.013012 | 0.004176 | 0.00368 | 0.003609 | 0.016921 | 0.00368 | -0.00088 | 0.010933 | 0.012597 | 0.006288 | -0.00039 | 0.005962 | -0.02669 | -0.01653 | 0.008347 | -0.01230 | 0.019559 | -0.00828 | 0.013524 | 0.011063 | -0.01160 |
| 1.75 | 0.005191 | 0.003845 | 0.001499 | 0.006208 | -0.00809 | -0.00552 | 0.010073 | 0.011755 | 0.016617 | 0.012858 | 0.007316 | 0.008942 | 0.013898 | 0.01544 | 0.008942 | -0.00026 | 0.025879 | 0.01028 | 0.005526 | -0.00043 | 0.035686 | 0.001279 | -0.02620 | 0.015594 | -0.00556 | 0.004173 | -0.01987 | 0.004814 | 0.001898 | -0.00851 |
| 2.00 | 0.008639 | 0.014003 | 0.003645 | 0.005898 | -0.00154 | -0.00153 | 0.013347 | 0.022035 | 0.017569 | 0.00768 | 0.008876 | 0.020467 | 0.01461 | 0.018084 | 0.020467 | -0.00156 | 0.025787 | 0.010512 | 0.011047 | -0.00011 | 0.03207 | -0.00379 | -0.01993 | 0.023312 | -0.00395 | 0.020537 | -0.02432 | 0.006494 | -0.00351 | -0.01210 |
| 2.25 | 0.01795 | 0.017649 | -0.00137 | 0.005692 | 0.002046 | -0.01921 | 0.019129 | 0.030364 | 0.026724 | 0.023214 | 0.012557 | 0.006436 | -0.00059 | 0.019953 | 0.006436 | 0.005566 | 0.015476 | 0.003516 | -0.00394 | -0.00805 | 0.056696 | 0.013076 | 0.060484 | 0.095784 | -0.00810 | 0.001835 | -0.00779 | 0.086452 | -0.00503 | 0.008253 |
| 2.50 | 0.017692 | 0.01642 | -0.00831 | 0.008276 | 0.001781 | -0.01960 | 0.023529 | 0.035851 | 0.046656 | 0.022209 | -0.01765 | -0.01524 | -0.01590 | -0.00959 | -0.01524 | -0.00859 | -0.00954 | -0.02308 | -0.03236 | -0.03350 | 0.683221 | 0.016063 | 0.808953 | 0.678477 | -0.02551 | 0.044894 | 0.124937 | 1.59090 | 0.014972 | 0.202988 |
| 2.75 | 0.019432 | 0.023137 | -0.00310 | 0.000317 | 0.006736 | -0.02288 | 0.010994 | 0.04038 | 0.052468 | 0.030053 | -0.02737 | -0.03006 | -0.01743 | -0.01847 | -0.03006 | -0.03590 | -0.02611 | -0.04059 | -0.03669 | -0.04585 | 3.230761 | 0.0000113 | 3.319382 | 1.711954 | -0.02296 | 0.210063 | 0.64953 | 2.64254 | 0.147394 | 1.465829 |
| 3.00 | 0.014696 | 0.035466 | -0.00363 | 0.017889 | -0.00446 | -0.00327 | 0.019282 | 0.058262 | 0.062447 | 0.031831 | -0.02721 | -0.03407 | -0.00661 | -0.03092 | -0.03407 | -0.02765 | -0.02869 | -0.04861 | -0.04892 | -0.05588 | 3.460383 | 0.001731 | 3.945991 | 2.128243 | -0.01261 | 0.925543 | 2.32625 | 2.774394 | 0.414244 | 2.830216 |
| 3.25 | 0.031449 | 0.021622 | 0.002993 | 0.010291 | 0.004966 | -0.00745 | 0.02665 | 0.061667 | 0.066999 | 0.028121 | -0.02627 | -0.03257 | 0.000654 | -0.02676 | -0.03257 | -0.04064 | -0.03406 | -0.04606 | -0.04863 | -0.04696 | 3.454887 | -0.00168 | 4.087781 | 2.295729 | 0.008046 | 2.022467 | 3.28824 | 2.808646 | 0.880843 | 3.119383 |
| 3.50 | 0.031321 | 0.019287 | 0.004985 | 0.008637 | 0.007134 | -0.00751 | 0.022096 | 0.055948 | 0.076026 | 0.034381 | -0.01085 | -0.03829 | 0.018597 | -0.01371 | -0.03829 | -0.03528 | -0.03041 | -0.04078 | -0.04178 | -0.04810 | 3.411675 | 0.030365 | 4.110719 | 2.383542 | 0.023373 | 2.361451 | 3.548262 | 2.837941 | 1.277906 | 3.131424 |
| 3.75 | 0.032126 | 0.031083 | 0.001997 | 0.013961 | -0.00348 | -0.01381 | 0.027827 | 0.06636 | 0.085079 | 0.038013 | 0.005856 | -0.04071 | 0.028708 | -0.00504 | -0.04071 | -0.03670 | -0.02301 | -0.03777 | -0.04159 | -0.04548 | 3.392075 | 0.011386 | 4.127513 | 2.417801 | 0.041719 | 2.470675 | 3.645385 | 2.849788 | 1.509974 | 3.124856 |
| 4.00 | 0.041856 | 0.02580 | 0.013836 | 0.021455 | 0.022222 | -0.00012 | 0.024654 | 0.072409 | 0.086854 | 0.047403 | 0.016377 | -0.03148 | 0.03978 | -0.00871 | -0.03148 | -0.03490 | -0.01985 | -0.03151 | -0.03197 | -0.03914 | 3.366618 | 0.019292 | 4.11961 | 2.449284 | 0.067161 | 2.534992 | 3.685193 | 2.839817 | 1.633206 | 3.120627 |
| 4.25 | 0.041631 | 0.034319 | 0.006288 | 0.023522 | 0.034256 | 0.009458 | 0.039083 | 0.086623 | 0.098967 | 0.047751 | 0.03338 | -0.02317 | 0.052917 | 0.005781 | -0.02317 | -0.03574 | -0.00659 | -0.02127 | -0.01846 | -0.02421 | 3.33241 | 0.04352 | 4.125623 | 2.447214 | 0.092288 | 2.564242 | 3.723974 | 2.853961 | 1.71503 | 3.12605 |
| 4.50 | 0.047462 | 0.035302 | 0.013261 | 0.024814 | 0.032309 | 0.009973 | 0.033608 | 0.090391 | 0.125508 | 0.058841 | 0.041641 | -0.01937 | 0.058293 | -0.00617 | -0.01937 | -0.04055 | 0.000959 | -0.01835 | -0.02769 | -0.02110 | 3.320368 | 0.030667 | 4.132926 | 2.473897 | 0.09660 | 2.578922 | 3.757375 | 2.863651 | 1.78180 | 3.092168 |
| 4.75 | 0.061413 | 0.042962 | 0.032149 | 0.026261 | 0.030053 | -0.00218 | 0.037241 | 0.103183 | 0.122422 | 0.058918 | 0.067764 | -0.01945 | 0.074848 | 0.014982 | -0.01945 | -0.01584 | 0.008846 | -0.01085 | -0.01222 | -0.02220 | 3.306844 | 0.067839 | 4.123862 | 2.491497 | 0.124041 | 2.595939 | 3.750529 | 2.853204 | 1.815946 | 3.089183 |
| 5.00 | 0.057579 | 0.03989 | 0.034793 | 0.041042 | 0.038681 | 0.005087 | 0.042408 | 0.105265 | 0.122937 | 0.064791 | 0.087067 | -0.00726 | 0.073281 | 0.028202 | -0.00726 | -0.02116 | 0.013266 | 0.004118 | -0.01232 | -0.01332 | 3.271117 | 0.04874 | 4.110375 | 2.497662 | 0.14466 | 2.603387 | 3.755468 | 2.841274 | 1.847731 | 3.069829 |
| 5.25 | 0.070885 | 0.037678 | 0.033988 | 0.037011 | 0.015408 | -0.00372 | 0.035706 | 0.119412 | 0.126074 | 0.070935 | 0.10819 | 0.004849 | 0.085706 | 0.022103 | 0.004849 | -0.01865 | 0.037388 | 0.01774 | -0.00028 | -0.00890 | 3.251409 | 0.078068 | 4.112737 | 2.505521 | 0.161125 | 2.612086 | 3.77366 | 2.850096 | 1.861923 | 3.089382 |
| 5.50 | 0.068984 | 0.043617 | 0.039697 | 0.033704 | 0.041026 | 0.001808 | 0.045478 | 0.132072 | 0.14490 | 0.071012 | 0.127172 | -0.00070 | 0.089943 | 0.040434 | -0.00070 | -0.01542 | 0.040703 | 0.024737 | -0.00061 | 0.00378 | 3.236764 | 0.072697 | 4.112651 | 2.520062 | 0.17853 | 2.623884 | 3.785153 | 2.828671 | 1.870402 | 3.059182 |
| 5.75 | 0.07372 | 0.046361 | 0.048164 | 0.026778 | 0.035407 | -0.00166 | 0.056171 | 0.134188 | 0.148166 | 0.07990 | 0.141714 | 0.008775 | 0.099413 | 0.04403 | 0.008775 | -0.00524 | 0.04902 | 0.035718 | 0.003385 | 0.000302 | 3.231701 | 0.06799 | 4.095513 | 2.51098 | 0.192564 | 2.61774 | 3.78349 | 2.849144 | 1.871723 | 3.051271 |
| 6.00 | 0.074107 | 0.050457 | 0.05667 | 0.038097 | 0.04837 | -0.00366 | 0.063437 | 0.140831 | 0.157785 | 0.080557 | 0.160037 | 0.015331 | 0.10429 | 0.047379 | 0.015331 | 0.002843 | 0.067219 | 0.048785 | 0.003052 | 0.005212 | 3.193371 | 0.087663 | 4.085204 | 2.51818 | 0.215457 | 2.598983 | 3.794053 | 2.836289 | 1.868118 | 3.040425 |
| 6.25 | 0.075525 | 0.048573 | 0.057244 | 0.050655 | 0.048237 | 0.003544 | 0.070702 | 0.154946 | 0.158068 | 0.09126 | 0.176499 | 0.024351 | 0.100623 | 0.054641 | 0.024351 | -0.00185 | 0.083239 | 0.043178 | 0.009572 | 0.008649 | 3.196372 | 0.092762 | 4.098477 | 2.497615 | 0.214594 | 2.601919 | 3.758305 | 2.836429 | 1.859816 | 3.036693 |
| 6.50 | 0.080293 | 0.059182 | 0.05552 | 0.053136 | 0.062838 | -0.01639 | 0.061185 | 0.172993 | 0.173164 | 0.096516 | 0.18290 | 0.02456 | 0.119848 | 0.079741 | 0.02456 | 0.007744 | 0.093735 | 0.057959 | 0.013284 | 0.017323 | 3.169613 | 0.100788 | 4.075797 | 2.489473 | 0.223807 | 2.598331 | 3.779382 | 2.810579 | 1.846919 | 3.024902 |
| 6.75 | 0.075299 | 0.05357 | 0.062378 | 0.051999 | 0.056157 | 0.001037 | 0.06579 | 0.182546 | 0.184377 | 0.10123 | 0.18682 | 0.030197 | 0.119136 | 0.085769 | 0.030197 | 0.008582 | 0.107178 | 0.057078 | 0.018471 | 0.023174 | 3.158656 | 0.102025 | 4.067335 | 2.489003 | 0.229961 | 2.590013 | 3.779773 | 2.821781 | 1.834657 | 2.982163 |
| 7.00 | 0.088734 | 0.074009 | 0.069389 | 0.052619 | 0.055493 | -0.01195 | 0.069985 | 0.195371 | 0.185355 | 0.107026 | 0.18548 | 0.035626 | 0.119848 | 0.088025 | 0.035626 | 0.01164 | 0.120282 | 0.063519 | 0.024277 | 0.025711 | 3.140648 | 0.10293 | 4.052817 | 2.482038 | 0.25356 | 2.58724 | 3.777866 | 2.786885 | 1.826279 | 2.971417 |
| 7.25 | 0.095436 | 0.081014 | 0.082148 | 0.05603 | 0.046512 | 0.001937 | 0.07193 | 0.194644 | 0.19770 | 0.111045 | 0.196762 | 0.043017 | 0.13060 | 0.083654 | 0.043017 | 0.014907 | 0.135382 | 0.08196 | 0.033128 | 0.024729 | 3.099244 | 0.138624 | 4.02451 | 2.459779 | 0.25748 | 2.591807 | 3.766814 | 2.78921 | 1.816428 | 2.949475 |
| 7.50 | 0.090023 | 0.081177 | 0.085251 | 0.063266 | 0.054653 | 0.000458 | 0.067018 | 0.20813 | 0.204772 | 0.117459 | 0.194981 | 0.051035 | 0.129532 | 0.097966 | 0.051035 | 0.018049 | 0.129305 | 0.08474 | 0.036698 | 0.033854 | 3.09071 | 0.128064 | 4.010164 | 2.461802 | 0.263007 | 2.57691 | 3.756691 | 2.78050 | 1.79721 | 2.93032 |
| 7.75 | 0.098883 | 0.089001 | 0.097779 | 0.069416 | 0.066333 | 0.01203 | 0.067427 | 0.218211 | 0.210687 | 0.124723 | 0.216244 | 0.059094 | 0.13886 | 0.098671 | 0.059094 | 0.023787 | 0.133326 | 0.10017 | 0.040933 | 0.033936 | 3.061456 | 0.124654 | 3.998566 | 2.443684 | 0.279707 | 2.577182 | 3.760652 | 2.784169 | 1.781165 | 2.903155 |
| 8.00 | 0.097627 | 0.090516 | 0.101879 | 0.076755 | 0.067439 | 0.009908 | 0.078734 | 0.231499 | 0.225012 | 0.137977 | 0.210283 | 0.05400 | 0.122376 | 0.101597 | 0.05400 | 0.022824 | 0.151464 | 0.109298 | 0.046216 | 0.044697 | 3.042543 | 0.126465 | 3.991436 | 2.427919 | 0.281628 | 2.576693 | 3.729256 | 2.755098 | 1.758976 | 2.882109 |
| 8.25 | 0.109354 | 0.094817 | 0.107204 | 0.074843 | 0.066422 | 0.006373 | 0.08513 | 0.244291 | 0.232085 | 0.139446 | 0.216524 | 0.064189 | 0.134445 | 0.098354 | 0.064189 | 0.039872 | 0.155975 | 0.112124 | 0.047786 | 0.05153 | 3.011011 | 0.124141 | 3.966566 | 2.425802 | 0.288684 | 2.559295 | 3.747008 | 2.749749 | 1.742144 | 2.87186 |
| 8.50 | 0.107389 | 0.103378 | 0.115327 | 0.074481 | 0.073811 | 0.032474 | 0.087279 | 0.256488 | 0.245253 | 0.151695 | 0.213524 | 0.067571 | 0.143666 | 0.106673 | 0.067571 | 0.039286 | 0.160916 | 0.124356 | 0.054354 | 0.048339 | 2.982588 | 0.12396 | 3.955484 | 2.40599 | 0.290761 | 2.552934 | 3.702065 | 2.734485 | 1.719549 | 2.837281 |
| 8.75 | 0.118504 | 0.112225 | 0.113986 | 0.08704 | 0.083987 | 0.011259 | 0.086614 | 0.268487 | 0.247722 | 0.15154 | 0.217944 | 0.066778 | 0.139821 | 0.112067 | 0.066778 | 0.030322 | 0.161346 | 0.125376 | 0.060302 | 0.052021 | 2.967075 | 0.163879 | 3.938302 | 2.411213 | 0.305383 | 2.548367 | 3.692871 | 2.717485 | 1.70523 | 2.830216 |
| 9.00 | 0.127139 | 0.101412 | 0.11774 | 0.099599 | 0.085137 | 0.00528 | 0.095618 | 0.280287 | 0.260375 | 0.168697 | 0.223205 | 0.074295 | 0.151427 | 0.112278 | 0.074295 | 0.044312 | 0.168834 | 0.138488 | 0.063538 | 0.059386 | 2.921585 | 0.148068 | 3.92881 | 2.404107 | 0.30209 | 2.541245 | 3.69380 | 2.707235 | 1.683423 | 2.79922 |
| 9.25 | 0.134678 | 0.134261 | 0.131993 | 0.095361 | 0.105003 | 0.01788 | 0.098177 | 0.299029 | 0.265133 | 0.172406 | 0.226806 | 0.082521 | 0.15513 | 0.114288 | 0.082521 | 0.049171 | 0.160579 | 0.145809 | 0.07101 | 0.062332 | 2.919668 | 0.159232 | 3.903037 | 2.383636 | 0.302012 | 2.523032 | 3.63981 | 2.680405 | 1.662173 | 2.787727 |
| 9.50 | 0.143248 | 0.116853 | 0.133678 | 0.094482 | 0.094827 | 0.020387 | 0.099609 | 0.306532 | 0.274211 | 0.176889 | 0.227626 | 0.088535 | 0.151819 | 0.122043 | 0.088535 | 0.052397 | 0.171105 | 0.154891 | 0.078815 | 0.065074 | 2.881519 | 0.14303 | 3.898527 | 2.384483 | 0.319495 | 2.517704 | 3.629883 | 2.656235 | 1.643158 | 2.744093 |
| 9.75 | 0.143571 | 0.131312 | 0.144828 | 0.105801 | 0.102923 | 0.032859 | 0.099865 | 0.318365 | 0.28275 | 0.188133 | 0.232786 | 0.085486 | 0.157728 | 0.12860 | 0.085486 | 0.064125 | 0.176998 | 0.161007 | 0.085715 | 0.071007 | 2.850095 | 0.164181 | 3.879284 | 2.37606 | 0.316203 | 2.502046 | 3.615994 | 2.640328 | 1.618329 | 2.732899 |
| 10.00 | 0.157328 | 0.123448 | 0.145211 | 0.11345 | 0.101154 | 0.024181 | 0.109381 | 0.340412 | 0.280127 | 0.20529 | 0.232346 | 0.088242 | 0.167554 | 0.125498 | 0.088242 | 0.068147 | 0.187064 | 0.169255 | 0.086572 | 0.068183 | 2.81632 | 0.161465 | 3.849302 | 2.373283 | 0.323729 | 2.514876 | 3.567873 | 2.627193 | 1.606879 | 2.712599 |
