# Supplementary material for: Neuronal junctophilins recruit specific CaV and RyR isoforms to ER-PM junctions and functionally alter CaV2.1 and CaV2.2
Source: eLife. 2021 Mar 26;10:e64249. doi: 10.7554/eLife.64249 (PMC8046434; doi:10.7554/eLife.64249)
Supplement: Figure 6—source data 1. [file elife-64249-fig6-data1.docx]

**Figure 6**

| **Cell** | **JPH3 vs RyR2**  **(with Ca_V_1.2)** | **JPH4 vs RyR3**  **(with Ca_V_1.2)** |
| --- | --- | --- |
| 1 | 0.58 | 0.60 |
| 2 | 0.85 | 0.50 |
| 3 | 0.58 | 0.21 |
| 4 | 0.57 | 0.42 |
| 5 | 0.36 | 0.26 |
| 6 | 0.49 | 0.68 |
| 7 | 0.70 | 0.65 |
| 8 | 0.36 | 0.55 |
| 9 | 0.47 | 0.63 |
| 10 | 0.46 | 0.76 |
| 11 | 0.55 | 0.72 |
| 12 | 0.31 | 0.76 |
| 13 | 0.35 | 0.64 |
| 14 | 0.36 | 0.59 |
| 15 | 0.39 | 0.72 |
| 16 | 0.45 |  |
| 17 | 0.48 |  |
| 18 | 0.39 |  |
| 19 | 0.63 |  |

**JPH3 vs RyR2 and JPH4 vs RyR3 with Ca_V_1.2 present**

(Pearson’s coefficients for JPH3 vs RyR2 and JPH4 vs RyR3 w/o Ca_V_1.2 are in “Figure 5-source data 1”)

**Pearson’s Coefficients**

**Statistics**

[JPH3 vs RyR2 (without Ca_V_1.2)] vs [JPH3 vs RyR2 (with Ca_V_1.2)]

**T-test with Welch’s correction:** p<0.469

[JPH4 vs RyR2 (without Ca_V_1.2)] vs [JPH4 vs RyR2 (with Ca_V_1.2)]

**T-test with Welch’s correction:** p<0.409
