# Supplementary material for: Neuronal junctophilins recruit specific CaV and RyR isoforms to ER-PM junctions and functionally alter CaV2.1 and CaV2.2
Source: eLife. 2021 Mar 26;10:e64249. doi: 10.7554/eLife.64249 (PMC8046434; doi:10.7554/eLife.64249)
Supplement: Figure 7—source data 1. [file elife-64249-fig7-data1.docx]

**Fig 7B Fig 7D**

|  | **Ca_V_2.1 vs RyR1…** | |
| --- | --- | --- |
| **Cell** | **+ JPH3** | **+ JPH4** |
| 1 | 0.60 | 0.45 |
| 2 | 0.50 | 0.33 |
| 3 | 0.80 | 0.42 |
| 4 | 0.53 | 0.39 |
| 5 | 0.60 | 0.34 |
| 6 | 0.60 | 0.56 |
| 7 | 0.55 | 0.29 |
| 8 | 0.54 | 0.57 |
| 9 | 0.71 | 0.45 |
| 10 | 0.47 | 0.31 |
| 11 | 0.80 | 0.03 |
| 12 | 0.65 | 0.24 |

**Pearson’s Coefficients Pearson’s Coefficients**

|  | **Ca_V_2.2 VS RyR1…** | |
| --- | --- | --- |
| **Cell** | **+ JPH3** | **+ JPH4** |
| 1 | 0.43 | 0.44 |
| 2 | 0.68 | 0.71 |
| 3 | 0.70 | 0.41 |
| 4 | 0.79 | 0.32 |
| 5 | 0.79 | 0.57 |
| 6 | 0.72 | 0.54 |
| 7 | 0.68 | 0.17 |
| 8 | 0.74 | 0.03 |
| 9 | 0.57 | 0.36 |
| 10 | 0.56 | 0.47 |
| 11 | 0.46 | 0.59 |
| 12 | 0.54 | 0.25 |
| 13 | 0.76 | 0.53 |
| 14 | 0.76 | 0.29 |
| 15 |  | 0.31 |

**Statistics Statistics**

[Ca_V_2.1 vs RyR1 with JPH3] vs [Ca_V_2.1 vs RyR1 with JPH4] [Ca_V_2.2 vs RyR1 with JPH3] vs [Ca_V_2.2 vs RyR1 with JPH4]

**T-test with Welch’s correction:** p < 0.0001 **T-test with Welch’s correction:** p = 0.0001
