# Supplementary material for: Neuronal junctophilins recruit specific CaV and RyR isoforms to ER-PM junctions and functionally alter CaV2.1 and CaV2.2
Source: eLife. 2021 Mar 26;10:e64249. doi: 10.7554/eLife.64249 (PMC8046434; doi:10.7554/eLife.64249)
Supplement: Figure 7—source data 2. [file elife-64249-fig7-data2.docx]

**Figure 7G ΔF/Fo vs time**

|  | **RyR1-stables + Ca_V_2.1 + β1b +α2δ1 + R-CEPIAer** | | | | | | | | |
| --- | --- | --- | --- | --- | --- | --- | --- | --- | --- |
| Time (s) | **Fluo-8 (ΔF/Fo)** | | | |  | **R-CEPIAer (ΔF/Fo)** | | | |
|  | **Cell1** | **Cell2*** | **Cell3** | **Cell4** |  | **Cell1** | **Cell2*** | **Cell3** | **Cell4** |
| 0.90 | 0.044137 | **-0.011360** | -0.039830 | 0.025376 |  | -0.028370 | **0.020067** | -0.022510 | 0.011292 |
| 1.05 | 0.081829 | **0.004555** | 0.015263 | 0.028528 |  | -0.022120 | **0.047232** | 0.007915 | -0.030820 |
| 1.20 | 0.063136 | **0.012155** | 0.035640 | 0.011579 |  | -0.047900 | **-0.026350** | -0.017940 | -0.002320 |
| 1.35 | 0.065128 | **0.001563** | 0.084439 | 0.012618 |  | -0.022770 | **0.008643** | -0.007850 | -0.017860 |
| 1.50 | 0.049959 | **-0.051760** | 0.034794 | 0.018663 |  | -0.025320 | **-0.006530** | -0.002740 | -0.003610 |
| 1.65 | 0.048733 | **-0.034400** | 0.061578 | -0.003440 |  | 0.009876 | **0.021956** | -0.020070 | -0.009800 |
| 1.80 | 0.033794 | **-0.020940** | 0.009674 | 0.012210 |  | -0.020970 | **0.006020** | -0.013430 | -0.006590 |
| 1.95 | 0.008436 | **-0.023450** | 0.014021 | 0.016326 |  | 0.000547 | **-0.026000** | -0.024200 | -0.024830 |
| 2.10 | 0.017169 | **-0.008550** | 0.010324 | -0.002480 |  | 0.018912 | **-0.014860** | 0.004585 | -0.008310 |
| 2.25 | 0.022532 | **0.002640** | 0.012723 | -0.002510 |  | 0.004847 | **0.041205** | -0.004120 | 0.024275 |
| 2.40 | 0.015867 | **-0.015910** | -0.013690 | -0.010710 |  | -0.000850 | **0.003606** | -0.017250 | -0.010080 |
| 2.55 | -0.003060 | **0.007727** | -0.021260 | 0.013322 |  | -0.010530 | **-0.011280** | 0.000954 | 0.010377 |
| 2.70 | -0.011100 | **0.042496** | 0.024831 | -0.010860 |  | 0.003331 | **-0.008040** | 0.036963 | 0.010028 |
| 2.85 | -0.048490 | **0.021730** | -0.026990 | -0.013570 |  | -0.003840 | **0.011282** | 0.005332 | 0.009636 |
| 3.00 | -0.035160 | **-0.005740** | -0.009630 | -0.001730 |  | 0.008549 | **-0.001930** | 0.011165 | -0.004500 |
| 3.15 | -0.030640 | **-0.025790** | 0.296060 | 0.179545 |  | -0.008300 | **0.002062** | -0.003770 | 0.017827 |
| 3.30 | 0.003686 | **0.062544** | 0.545727 | 0.366240 |  | -0.018560 | **0.000128** | -0.018030 | 0.035537 |
| 3.45 | 0.093857 | **0.698695** | 0.800587 | 0.495599 |  | -0.000880 | **-0.009050** | -0.004890 | 0.061962 |
| 3.60 | 0.093398 | **0.851359** | 0.902983 | 0.595140 |  | -0.027000 | **-0.010500** | 0.014351 | -0.014460 |
| 3.75 | 0.136607 | **0.934902** | 1.003515 | 0.649658 |  | -0.005400 | **-0.020890** | 0.008924 | 0.020702 |
| 3.90 | 0.176138 | **1.035800** | 1.169189 | 0.711296 |  | -0.025630 | **-0.001480** | -0.000750 | 0.001380 |
| 4.05 | 0.198815 | **1.088105** | 1.225382 | 0.773082 |  | -0.006100 | **-0.074230** | -0.010820 | 0.004495 |
| 4.20 | 0.223407 | **1.130954** | 1.269750 | 0.827860 |  | -0.020760 | **-0.099280** | -0.006850 | -0.004850 |
| 4.35 | 0.285616 | **1.173982** | 1.341805 | 0.861979 |  | 0.004206 | **-0.114680** | -0.003690 | -0.024280 |
| 4.50 | 0.271443 | **1.173922** | 1.401470 | 0.869582 |  | -0.005970 | **-0.119130** | 0.014023 | -0.015920 |
| 4.65 | 0.329284 | **1.203545** | 1.466299 | 0.879039 |  | -0.038330 | **-0.148230** | 0.012765 | -0.038860 |
| 4.80 | 0.333498 | **1.159440** | 1.549221 | 0.918611 |  | -0.017210 | **-0.159040** | 0.029399 | -0.033190 |
| 4.95 | 0.329897 | **1.200015** | 1.544423 | 0.929811 |  | -0.006450 | **-0.175060** | 0.017588 | -0.074450 |
| 5.10 | 0.341925 | **1.339692** | 1.578263 | 0.927734 |  | -0.032980 | **-0.204000** | -0.022890 | -0.077480 |
| 5.25 | 0.383142 | **1.227603** | 1.674929 | 0.935523 |  | -0.010030 | **-0.221970** | 0.023841 | -0.089610 |
| 5.40 | 0.432863 | **1.272187** | 1.621981 | 0.941864 |  | -0.037010 | **-0.187240** | -0.022800 | -0.073410 |
| 5.55 | 0.424665 | **1.221798** | 1.663781 | 0.957775 |  | -0.022640 | **-0.237010** | -0.012770 | -0.058050 |
| 5.70 | 0.411412 | **1.227064** | 1.695165 | 0.931962 |  | -0.025400 | **-0.231520** | 0.006840 | -0.084650 |
| 5.85 | 0.419916 | **1.276616** | 1.719240 | 0.936227 |  | -0.041230 | **-0.232820** | 0.009723 | -0.093340 |
| 6.00 | 0.412944 | **1.299955** | 1.758273 | 0.933483 |  | -0.057450 | **-0.199690** | 0.011532 | -0.115650 |
| 6.15 | 0.447189 | **1.255551** | 1.759007 | 0.924582 |  | -0.058150 | **-0.222770** | -0.013990 | -0.119310 |
| 6.30 | 0.462971 | **1.222815** | 1.847291 | 0.916831 |  | -0.029800 | **-0.207230** | 0.030186 | -0.093750 |
| 6.45 | 0.455080 | **1.272008** | 1.824881 | 0.934336 |  | -0.060130 | **-0.227770** | -0.004450 | -0.070880 |
| 6.60 | 0.504188 | **1.279848** | 1.855335 | 0.904629 |  | -0.059390 | **-0.223590** | -0.016650 | -0.133070 |
| 6.75 | 0.476991 | **1.233228** | 1.848618 | 0.927215 |  | -0.046690 | **-0.215690** | 0.005870 | -0.122770 |
| 6.90 | 0.498595 | **1.241248** | 1.854996 | 0.936153 |  | -0.042410 | **-0.249090** | -0.032250 | -0.128850 |
| 7.05 | 0.484499 | **1.176196** | 1.888018 | 0.901625 |  | -0.073160 | **-0.208930** | 0.006984 | -0.126800 |
| 7.20 | 0.483580 | **1.302529** | 1.925075 | 0.905594 |  | -0.056760 | **-0.215000** | -0.047490 | -0.097280 |
| 7.35 | 0.527018 | **1.335982** | 1.910512 | 0.895617 |  | -0.090140 | **-0.208120** | -0.029230 | -0.117560 |
| 7.50 | 0.514071 | **1.253157** | 1.965633 | 0.914420 |  | -0.080610 | **-0.212770** | -0.051020 | -0.130390 |
| 7.65 | 0.524873 | **1.237896** | 1.901622 | 0.866170 |  | -0.070480 | **-0.236620** | -0.034920 | -0.108240 |
| 7.80 | 0.524490 | **1.225509** | 1.992276 | 0.903517 |  | -0.087520 | **-0.234010** | -0.039380 | -0.126690 |
| 7.95 | 0.534526 | **1.217310** | 1.928152 | 0.894060 |  | -0.044830 | **-0.232000** | -0.024500 | -0.092950 |
| 8.10 | 0.539353 | **1.181403** | 1.965492 | 0.894616 |  | -0.105820 | **-0.236540** | -0.073120 | -0.141150 |
| 8.25 | 0.507559 | **1.234605** | 2.000122 | 0.895617 |  | -0.063670 | **-0.218930** | -0.031100 | -0.089740 |
| 8.40 | 0.502886 | **1.219404** | 1.981890 | 0.879929 |  | -0.072460 | **-0.252180** | -0.049510 | -0.085320 |
| 8.55 | 0.524490 | **1.139033** | 1.954456 | 0.873143 |  | -0.094850 | **-0.234080** | -0.052580 | -0.138430 |
| 8.70 | 0.560957 | **1.182121** | 2.044208 | 0.864205 |  | -0.113370 | **-0.212510** | -0.043810 | -0.138850 |
| 8.85 | 0.520353 | **1.167758** | 2.005061 | 0.857677 |  | -0.136770 | **-0.231040** | -0.038360 | -0.099530 |
| 9.00 | 0.526635 | **1.048847** | 2.033454 | 0.856713 |  | -0.090210 | **-0.239390** | -0.049300 | -0.137210 |
| 9.15 | 0.502426 | **1.126166** | 2.040341 | 0.863685 |  | -0.096860 | **-0.213170** | -0.040060 | -0.125990 |
| 9.30 | 0.495148 | **1.148309** | 2.064980 | 0.842212 |  | -0.108840 | **-0.234800** | -0.045720 | -0.115490 |
| 9.45 | 0.509474 | **1.069254** | 2.057473 | 0.826858 |  | -0.107910 | **-0.212660** | -0.063000 | -0.147760 |
| 9.60 | 0.515833 | **1.018445** | 2.110251 | 0.833719 |  | -0.082200 | **-0.211510** | -0.062730 | -0.099350 |
| 9.75 | 0.522881 | **1.058063** | 2.086205 | 0.857010 |  | -0.130120 | **-0.190170** | -0.075060 | -0.111790 |
| 9.90 | 0.510547 | **1.058482** | 2.086854 | 0.839134 |  | -0.108330 | **-0.222890** | -0.098170 | -0.111270 |
| 10.05 | 0.475306 | **0.954531** | 2.043982 | 0.802974 |  | -0.118330 | **-0.221030** | -0.090280 | -0.153090 |
| 10.20 | 0.520200 | **0.999056** | 2.125125 | 0.806127 |  | -0.084280 | **-0.224280** | -0.074130 | -0.141790 |
| 10.35 | 0.470862 | **0.954651** | 2.057191 | 0.794815 |  | -0.117310 | **-0.181030** | -0.074330 | -0.095500 |
| 10.50 | 0.515833 | **0.989122** | 2.077568 | 0.783949 |  | -0.091440 | **-0.208990** | -0.087210 | -0.111480 |
| 10.65 | 0.522958 | **0.972784** | 2.111945 | 0.810021 |  | -0.094030 | **-0.206470** | -0.094020 | -0.132420 |
| 10.80 | 0.508861 | **0.989600** | 2.122359 | 0.788733 |  | -0.119150 | **-0.200100** | -0.084080 | -0.095060 |
| 10.95 | 0.523341 | **0.971767** | 2.115642 | 0.789326 |  | -0.107530 | **-0.203010** | -0.092990 | -0.110980 |
| 11.10 | 0.479826 | **0.915872** | 2.054792 | 0.773565 |  | -0.103840 | **-0.184160** | -0.074310 | -0.120850 |
| 11.25 | 0.495225 | **0.925686** | 2.141156 | 0.782354 |  | -0.110420 | **-0.206060** | -0.095330 | -0.121200 |
| 11.40 | 0.433552 | **0.908810** | 2.069411 | 0.775493 |  | -0.118040 | **-0.184160** | -0.079690 | -0.134210 |
| 11.55 | 0.464503 | **0.877631** | 2.102603 | 0.751350 |  | -0.100870 | **-0.183010** | -0.089850 | -0.122310 |
| 11.70 | 0.487257 | **0.920300** | 2.067577 | 0.767816 |  | -0.121260 | **-0.194300** | -0.105280 | -0.123990 |
| 11.85 | 0.472471 | **0.849204** | 2.060154 | 0.783578 |  | -0.122600 | **-0.167250** | -0.124190 | -0.163220 |
| 12.00 | 0.428113 | **0.872664** | 2.106328 | 0.754316 |  | -0.113590 | **-0.170860** | -0.131130 | -0.119150 |
| 12.15 | 0.481664 | **0.823172** | 2.052759 | 0.738703 |  | -0.135570 | **-0.142650** | -0.084280 | -0.142240 |
| 12.30 | 0.436847 | **0.858121** | 2.107880 | 0.723089 |  | -0.118010 | **-0.178830** | -0.118750 | -0.093490 |
| 12.45 | 0.434702 | **0.743040** | 2.098623 | 0.708626 |  | -0.138080 | **-0.168630** | -0.099270 | -0.120240 |
| 12.60 | 0.449334 | **0.740706** | 2.112932 | 0.722311 |  | -0.123930 | **-0.168960** | -0.106990 | -0.128800 |
| 12.75 | 0.390650 | **0.749264** | 2.064783 | 0.745638 |  | -0.076340 | **-0.153440** | -0.089890 | -0.128430 |
| 12.90 | 0.417004 | **0.740586** | 2.053606 | 0.695831 |  | -0.123800 | **-0.130520** | -0.123640 | -0.149390 |
| 13.05 | 0.403138 | **0.690257** | 2.028318 | 0.701282 |  | -0.107180 | **-0.196020** | -0.077660 | -0.149520 |
| 13.20 | 0.416468 | **0.664284** | 2.086797 | 0.706512 |  | -0.120910 | **-0.145800** | -0.084010 | -0.187190 |
| 13.35 | 0.452782 | **0.670209** | 2.070174 | 0.703285 |  | -0.109290 | **-0.155020** | -0.111060 | -0.143380 |
| 13.50 | 0.399614 | **0.670687** | 2.044292 | 0.705325 |  | -0.122850 | **-0.135230** | -0.106810 | -0.127950 |
| 13.65 | 0.403597 | **0.628856** | 1.988297 | 0.672948 |  | -0.115360 | **-0.132490** | -0.084390 | -0.139980 |
| 13.80 | 0.441137 | **0.702525** | 2.071359 | 0.685891 |  | -0.087630 | **-0.120990** | -0.111620 | -0.131570 |
| 13.95 | 0.382223 | **0.644954** | 2.075197 | 0.658892 |  | -0.118800 | **-0.121900** | -0.120310 | -0.146030 |
| 14.10 | 0.367896 | **0.534241** | 2.031789 | 0.642611 |  | -0.119880 | **-0.114840** | -0.134230 | -0.148670 |
| 14.25 | 0.405053 | **0.514971** | 2.001872 | 0.657446 |  | -0.125680 | **-0.115920** | -0.104020 | -0.180300 |
| 14.40 | 0.375634 | **0.485168** | 1.942969 | 0.652662 |  | -0.070320 | **-0.073960** | -0.103030 | -0.158390 |
| 14.55 | 0.400533 | **0.501925** | 1.994986 | 0.655814 |  | -0.140890 | **-0.077810** | -0.131850 | -0.129760 |
| 14.70 | 0.387586 | **0.475174** | 1.949715 | 0.639607 |  | -0.126840 | **-0.097800** | -0.130330 | -0.152980 |
| 14.85 | 0.382070 | **0.398334** | 2.005203 | 0.641610 |  | -0.103220 | **-0.109610** | -0.148700 | -0.147580 |
| 15.00 | 0.356711 | **0.431009** | 1.957025 | 0.581418 |  | -0.103430 | **-0.077960** | -0.113950 | -0.164030 |
| 15.15 | 0.338095 | **0.419040** | 1.924963 | 0.603596 |  | -0.133680 | **-0.107530** | -0.122150 | -0.158100 |
| 15.30 | 0.378469 | **0.384091** | 1.930240 | 0.609418 |  | -0.109770 | **-0.098530** | -0.123390 | -0.125400 |
| 15.45 | 0.403751 | **0.320595** | 1.900690 | 0.610679 |  | -0.142670 | **-0.089610** | -0.128070 | -0.138150 |
| 15.60 | 0.344300 | **0.345191** | 1.892167 | 0.587723 |  | -0.135000 | **-0.090320** | -0.121730 | -0.111570 |
| 15.75 | 0.363376 | **0.358238** | 1.902976 | 0.588056 |  | -0.135330 | **-0.091740** | -0.115210 | -0.134290 |
| 15.90 | 0.344070 | **0.284808** | 1.849888 | 0.580639 |  | -0.103570 | **-0.096780** | -0.070290 | -0.159650 |
| 16.05 | 0.358014 | **0.323348** | 1.878930 | 0.572183 |  | -0.137770 | **-0.089070** | -0.152090 | -0.132220 |
| 16.20 | 0.324611 | **0.321613** | 1.824430 | 0.546705 |  | -0.098940 | **-0.094810** | -0.121230 | -0.118910 |
| 16.35 | 0.358703 | **0.267992** | 1.855504 | 0.562467 |  | -0.136530 | **-0.068610** | -0.088900 | -0.139930 |
| 16.50 | 0.340623 | **0.268590** | 1.799678 | 0.574631 |  | -0.117000 | **-0.058670** | -0.102640 | -0.100270 |
| 16.65 | 0.325837 | **0.222330** | 1.784973 | 0.547261 |  | -0.124850 | **-0.065700** | -0.122490 | -0.114080 |
| 16.80 | 0.306454 | **0.180439** | 1.763495 | 0.534058 |  | -0.140860 | **-0.064710** | -0.119390 | -0.126100 |
| 16.95 | 0.317563 | **0.166495** | 1.790561 | 0.527902 |  | -0.125630 | **-0.039670** | -0.101580 | -0.126520 |
| 17.10 | 0.296341 | **0.138966** | 1.759656 | 0.512028 |  | -0.100140 | **-0.048610** | -0.075780 | -0.122420 |
| 17.25 | 0.334417 | **0.152192** | 1.776083 | 0.527902 |  | -0.103400 | **-0.074540** | -0.103350 | -0.143590 |
| 17.40 | 0.296495 | **0.055902** | 1.721300 | 0.504871 |  | -0.110340 | **-0.049490** | -0.112180 | -0.147910 |
| 17.55 | 0.338018 | **0.113831** | 1.714527 | 0.523451 |  | -0.128020 | **-0.089690** | -0.085460 | -0.142720 |
| 17.70 | 0.285539 | **0.105274** | 1.757257 | 0.531907 |  | -0.103060 | **-0.068080** | -0.118210 | -0.147690 |
| 17.85 | 0.305765 | **0.090312** | 1.739110 | 0.512548 |  | -0.148170 | **-0.069520** | -0.114110 | -0.145790 |
| 18.00 | 0.270370 | **0.051413** | 1.713285 | 0.534689 |  | -0.111030 | **-0.039720** | -0.121390 | -0.127870 |
| 18.15 | 0.247540 | **0.076069** | 1.735723 | 0.505798 |  | -0.100290 | **-0.048820** | -0.112290 | -0.093030 |
| 18.30 | 0.260411 | **0.068170** | 1.676001 | 0.513178 |  | -0.086510 | **-0.039390** | -0.088790 | -0.143680 |
| 18.45 | 0.265390 | **0.078762** | 1.644024 | 0.499790 |  | -0.110420 | **-0.032480** | -0.098850 | -0.122360 |
| 18.60 | 0.257806 | **0.009642** | 1.653959 | 0.537173 |  | -0.108580 | **-0.031620** | -0.100560 | -0.125140 |
| 18.75 | 0.268685 | **0.084328** | 1.630928 | 0.498974 |  | -0.103480 | **-0.040860** | -0.102500 | -0.106470 |
| 18.90 | 0.260870 | **0.067153** | 1.596072 | 0.509915 |  | -0.123110 | **-0.047420** | -0.075760 | -0.107260 |
| 19.05 | 0.263858 | **0.001204** | 1.596552 | 0.500754 |  | -0.108430 | **-0.047600** | -0.101960 | -0.155250 |
| 19.20 | 0.270753 | **0.008265** | 1.608829 | 0.488701 |  | -0.123770 | **-0.014910** | -0.127150 | -0.167710 |
| 19.35 | 0.286382 | **0.011976** | 1.573182 | 0.511954 |  | -0.095230 | **-0.062370** | -0.091330 | -0.150350 |
| 19.50 | 0.268838 | **-0.011960** | 1.551337 | 0.511732 |  | -0.097870 | **-0.037820** | -0.090390 | -0.140920 |
| 19.65 | 0.217125 | **-0.035540** | 1.504317 | 0.498084 |  | -0.097390 | **-0.051650** | -0.124220 | -0.169690 |
| 19.80 | 0.258342 | **0.045668** | 1.559748 | 0.501570 |  | -0.094600 | **-0.026060** | -0.122420 | -0.122590 |
| 19.95 | 0.223024 | **-0.000890** | 1.523565 | 0.496118 |  | -0.114960 | **-0.025360** | -0.068490 | -0.130700 |
| 20.10 | 0.218811 | **-0.049370** | 1.459441 | 0.479392 |  | -0.118690 | **-0.039460** | -0.113250 | -0.143160 |
| 20.25 | 0.232601 | **-0.000830** | 1.505728 | 0.473718 |  | -0.131360 | **-0.052460** | -0.075490 | -0.146470 |
| 20.40 | 0.198126 | **-0.004780** | 1.389870 | 0.459476 |  | -0.105910 | **-0.038340** | -0.106220 | -0.135270 |
| 20.55 | 0.202492 | **0.013113** | 1.433136 | 0.493374 |  | -0.133340 | **-0.018010** | -0.096700 | -0.156880 |
| 20.70 | 0.233443 | **0.039684** | 1.406155 | 0.476870 |  | -0.105490 | **-0.046350** | -0.109790 | -0.146450 |
| 20.85 | 0.225169 | **0.011497** | 1.412025 | 0.465225 |  | -0.091760 | **-0.008780** | -0.101990 | -0.145990 |
| 21.00 | 0.230379 | **-0.021900** | 1.333112 | 0.456880 |  | -0.116840 | **-0.009920** | -0.094290 | -0.143830 |
| 21.15 | 0.173227 | **-0.008250** | 1.318294 | 0.452764 |  | -0.095020 | **-0.035750** | -0.090870 | -0.139500 |
| 21.30 | 0.175908 | **-0.008670** | 1.309234 | 0.464965 |  | -0.082210 | **-0.047450** | -0.072520 | -0.136800 |
| 21.45 | 0.198892 | **0.016644** | 1.217169 | 0.449129 |  | -0.100400 | **-0.026990** | -0.071400 | -0.173240 |
| 21.60 | 0.171695 | **-0.005500** | 1.236982 | 0.437744 |  | -0.112110 | **-0.026170** | -0.100560 | -0.139590 |
| 21.75 | 0.197513 | **-0.045890** | 1.211270 | 0.448870 |  | -0.105820 | **-0.047960** | -0.106140 | -0.136600 |
| 21.90 | 0.162348 | **0.023586** | 1.178107 | 0.419126 |  | -0.103960 | **-0.025330** | -0.059820 | -0.109130 |
| 22.05 | 0.183799 | **-0.010770** | 1.121265 | 0.454284 |  | -0.079400 | **-0.010080** | -0.100270 | -0.124770 |
| 22.20 | 0.158517 | **-0.026440** | 1.162443 | 0.431698 |  | -0.098650 | **-0.016140** | -0.063260 | -0.146010 |
| 22.35 | 0.192150 | **-0.038290** | 1.128913 | 0.430808 |  | -0.088410 | **-0.000130** | -0.073230 | -0.155710 |
| 22.50 | 0.163421 | **-0.025430** | 1.137578 | 0.410893 |  | -0.063150 | **-0.016380** | -0.066370 | -0.144570 |
| 22.65 | 0.175449 | **0.013053** | 1.088187 | 0.423280 |  | -0.095130 | **0.015480** | -0.089050 | -0.150850 |
| 22.80 | 0.199581 | **-0.033030** | 1.046782 | 0.410225 |  | -0.126390 | **-0.000350** | -0.065410 | -0.166730 |
| 22.95 | 0.154917 | **0.030408** | 1.008088 | 0.375920 |  | -0.125650 | **-0.016870** | -0.112040 | -0.138150 |
| 23.10 | 0.171082 | **-0.049660** | 0.952318 | 0.403290 |  | -0.102870 | **-0.033320** | -0.099990 | -0.109200 |
| 23.25 | 0.121821 | **0.025381** | 0.908543 | 0.376291 |  | -0.111280 | **-0.010710** | -0.102470 | -0.170130 |
| 23.40 | 0.162501 | **-0.013940** | 0.858135 | 0.390829 |  | -0.087910 | **-0.004770** | -0.111480 | -0.154570 |
| 23.55 | 0.130784 | **-0.016270** | 0.829488 | 0.358118 |  | -0.080640 | **-0.029110** | -0.073450 | -0.166210 |
| 23.70 | 0.157138 | **-0.036860** | 0.806570 | 0.367019 |  | -0.101990 | **-0.018240** | -0.059870 | -0.157990 |
| 23.85 | 0.134845 | **0.018199** | 0.820174 | 0.375327 |  | -0.113000 | **-0.009920** | -0.063200 | -0.129870 |
| 24.00 | 0.128026 | **-0.002510** | 0.755796 | 0.363533 |  | -0.091900 | **-0.027230** | -0.059330 | -0.136320 |
| 24.15 | 0.135228 | **0.014968** | 0.726726 | 0.389123 |  | -0.103270 | **0.009348** | -0.074900 | -0.145510 |
| 24.30 | 0.170699 | **0.022927** | 0.688962 | 0.393870 |  | -0.116710 | **-0.015130** | -0.049250 | -0.130150 |
| 24.45 | 0.104123 | **0.039025** | 0.642534 | 0.367019 |  | -0.081980 | **0.010937** | -0.081090 | -0.175400 |
| 24.60 | 0.088878 | **0.001742** | 0.643889 | 0.395168 |  | -0.104990 | **-0.017890** | -0.068100 | -0.172220 |
| 24.75 | 0.135534 | **0.071282** | 0.581515 | 0.366648 |  | -0.083540 | **-0.009380** | -0.048180 | -0.153900 |
| 24.90 | 0.095773 | **0.031006** | 0.566754 | 0.373732 |  | -0.092880 | **-0.006620** | -0.038580 | -0.140720 |
| 25.05 | 0.087422 | **-0.005860** | 0.491396 | 0.388047 |  | -0.109320 | **0.004506** | -0.075940 | -0.187930 |
| 25.20 | 0.083745 | **0.031006** | 0.491171 | 0.377626 |  | -0.088760 | **-0.015100** | -0.093870 | -0.183370 |
| 25.35 | 0.124732 | **0.055363** | 0.477313 | 0.385451 |  | -0.107400 | **0.008509** | -0.072160 | -0.113290 |
| 25.50 | 0.102361 | **0.048601** | 0.446323 | 0.375512 |  | -0.072870 | **-0.026980** | -0.061450 | -0.160540 |
| 25.65 | 0.060915 | **0.020234** | 0.459363 | 0.364905 |  | -0.093950 | **-0.018000** | -0.063330 | -0.135580 |
| 25.80 | 0.062294 | **0.019217** | 0.376752 | 0.375438 |  | -0.088830 | **-0.007310** | -0.042670 | -0.130590 |
| 25.95 | 0.094777 | **0.017661** | 0.380647 | 0.382633 |  | -0.095610 | **0.005000** | -0.113440 | -0.137170 |
| 26.10 | 0.100369 | **-0.010890** | 0.366845 | 0.376328 |  | -0.102890 | **-0.026840** | -0.022940 | -0.163310 |

***used for representative trace in Figure 7F**

**Figure 7J**

|  | **RyR1-stables + Ca_V_2.2 + β1b +α2δ1 + R-CEPIAer** | | | | | | | | |
| --- | --- | --- | --- | --- | --- | --- | --- | --- | --- |
| Time (s) | **Fluo-8 (ΔF/Fo)** | | | |  | **R-CEPIAer (ΔF/Fo)** | | | |
|  | **Cell1** | **Cell2** | **Cell3*** | **Cell4** |  | **Cell1** | **Cell2** | **Cell3*** | **Cell4** |
| 0.90 | 0.039155 | 0.032961 | **0.001817** | -0.042620 |  | -0.017220 | 0.010520 | **0.001070** | -0.011410 |
| 1.05 | 0.032003 | 0.019910 | **0.013864** | -0.022000 |  | -0.014070 | -0.035000 | **-0.007380** | 0.019137 |
| 1.20 | 0.029783 | -0.019540 | **0.020714** | -0.000120 |  | -0.035790 | -0.015650 | **0.018438** | 0.011848 |
| 1.35 | 0.009087 | -0.001840 | **0.033986** | -0.031700 |  | -0.040850 | 0.005536 | **0.003520** | -0.011590 |
| 1.50 | 0.006222 | -0.009270 | **0.006980** | -0.024000 |  | 0.007592 | 0.025360 | **-0.035400** | 0.025528 |
| 1.65 | -0.008210 | 0.010176 | **0.014360** | -0.013380 |  | 0.009162 | -0.012120 | **0.006300** | 0.041417 |
| 1.80 | 0.017908 | -0.015900 | **-0.014400** | -0.032460 |  | 0.005784 | -0.009930 | **0.015681** | 0.002862 |
| 1.95 | 0.001081 | 0.001770 | **0.036567** | -0.050550 |  | -0.021160 | 0.008326 | **0.027854** | 0.027764 |
| 2.10 | -0.017870 | 0.002605 | **-0.021650** | -0.030800 |  | 0.032287 | 0.015420 | **0.004167** | -0.019560 |
| 2.25 | 0.053838 | -0.008630 | **0.015849** | -0.017740 |  | 0.019939 | 0.003939 | **-0.011750** | -0.012420 |
| 2.40 | -0.007610 | 0.006243 | **0.020417** | -0.009650 |  | 0.010280 | -0.007680 | **-0.020380** | 0.004339 |
| 2.55 | 0.017358 | -0.012810 | **0.014327** | 0.066303 |  | -0.030900 | -0.023440 | **0.015988** | 0.013132 |
| 2.70 | -0.016200 | 0.028144 | **-0.004540** | 0.015271 |  | -0.027120 | 0.006730 | **-0.006660** | -0.018910 |
| 2.85 | -0.016200 | -0.003100 | **-0.012220** | 0.020934 |  | 0.003690 | 0.017308 | **-0.034450** | -0.009230 |
| 3.00 | -0.032310 | 0.001671 | **-0.034360** | 0.038688 |  | 0.007199 | -0.010680 | **0.009544** | 0.012014 |
| 3.15 | 0.032420 | 0.064571 | **0.350483** | 0.039355 |  | 0.033512 | -0.082280 | **-0.037360** | -0.046090 |
| 3.30 | 0.102971 | 0.080007 | **0.586061** | 0.040154 |  | 0.018536 | -0.032830 | **-0.019380** | -0.000530 |
| 3.45 | 0.159787 | 0.129068 | **0.720728** | 0.065970 |  | -0.061630 | 0.026942 | **0.048908** | -0.061690 |
| 3.60 | 0.199435 | 0.121325 | **0.747503** | 0.064038 |  | -0.033470 | -0.017330 | **-0.020380** | -0.030200 |
| 3.75 | 0.266969 | 0.151976 | **0.897196** | 0.056210 |  | -0.026540 | -0.017160 | **-0.013730** | 0.033162 |
| 3.90 | 0.274500 | 0.178842 | **0.926685** | 0.070833 |  | 0.056172 | 0.011714 | **-0.009650** | -0.029770 |
| 4.05 | 0.257579 | 0.209665 | **0.895509** | 0.073298 |  | -0.038670 | -0.058290 | **-0.058380** | -0.009850 |
| 4.20 | 0.309387 | 0.180882 | **0.994267** | 0.058508 |  | 0.007366 | -0.010800 | **-0.039360** | -0.030780 |
| 4.35 | 0.278825 | 0.230779 | **0.987846** | 0.057276 |  | 0.036105 | -0.044650 | **-0.077010** | -0.036380 |
| 4.50 | 0.331032 | 0.193688 | **0.982352** | 0.081559 |  | 0.038508 | -0.018060 | **-0.055160** | -0.067390 |
| 4.65 | 0.358349 | 0.230951 | **0.974575** | 0.050247 |  | -0.020740 | -0.026800 | **-0.052270** | -0.054940 |
| 4.80 | 0.395341 | 0.233335 | **1.080681** | 0.052879 |  | -0.040920 | -0.038940 | **-0.066710** | -0.051540 |
| 4.95 | 0.407728 | 0.225371 | **1.045897** | 0.060873 |  | -0.003030 | -0.067400 | **-0.075820** | -0.047400 |
| 5.10 | 0.419737 | 0.221905 | **1.092231** | 0.040787 |  | -0.040000 | -0.067500 | **-0.074020** | -0.072920 |
| 5.25 | 0.442937 | 0.184200 | **1.039178** | 0.066403 |  | -0.022650 | -0.047260 | **-0.103150** | -0.044610 |
| 5.40 | 0.476344 | 0.243708 | **1.045036** | 0.076362 |  | 0.017750 | -0.080560 | **-0.075830** | -0.015170 |
| 5.55 | 0.487745 | 0.249263 | **1.064728** | 0.064038 |  | -0.003680 | -0.080550 | **-0.107770** | -0.070010 |
| 5.70 | 0.521626 | 0.254203 | **1.091437** | 0.085390 |  | -0.053350 | -0.100650 | **-0.102710** | -0.043770 |
| 5.85 | 0.488637 | 0.229968 | **1.044275** | 0.059141 |  | -0.038590 | -0.064720 | **-0.107770** | -0.058620 |
| 6.00 | 0.522157 | 0.250049 | **1.059201** | 0.085356 |  | -0.044390 | -0.078130 | **-0.131050** | -0.012410 |
| 6.15 | 0.535474 | 0.201185 | **1.040668** | 0.059774 |  | 0.007794 | -0.106450 | **-0.158220** | -0.057090 |
| 6.30 | 0.561502 | 0.195163 | **1.097891** | 0.067335 |  | -0.051520 | -0.108850 | **-0.126660** | -0.059170 |
| 6.45 | 0.609781 | 0.222864 | **1.110666** | 0.096782 |  | -0.027710 | -0.098550 | **-0.118280** | -0.044040 |
| 6.60 | 0.604564 | 0.250270 | **1.098387** | 0.046117 |  | -0.062450 | -0.110260 | **-0.140670** | -0.059550 |
| 6.75 | 0.559206 | 0.252065 | **1.058606** | 0.061173 |  | -0.071700 | -0.094720 | **-0.136340** | -0.109310 |
| 6.90 | 0.623535 | 0.257005 | **1.063702** | 0.056443 |  | -0.122910 | -0.126620 | **-0.121430** | -0.084670 |
| 7.05 | 0.625526 | 0.220209 | **1.019321** | 0.060240 |  | -0.062220 | -0.057310 | **-0.141480** | -0.080020 |
| 7.20 | 0.641822 | 0.213868 | **1.063372** | 0.059874 |  | -0.087170 | -0.096190 | **-0.184110** | -0.063370 |
| 7.35 | 0.653906 | 0.192139 | **1.050299** | 0.052479 |  | -0.091960 | -0.085820 | **-0.176160** | -0.087030 |
| 7.50 | 0.656695 | 0.215269 | **0.999562** | 0.048448 |  | -0.119840 | -0.127040 | **-0.145190** | -0.084050 |
| 7.65 | 0.640949 | 0.193147 | **1.040337** | 0.008742 |  | -0.081750 | -0.135440 | **-0.162040** | -0.063630 |
| 7.80 | 0.674887 | 0.202586 | **0.976859** | 0.044318 |  | -0.074050 | -0.094240 | **-0.188820** | -0.060900 |
| 7.95 | 0.650207 | 0.193590 | **0.980367** | 0.046983 |  | -0.116230 | -0.102690 | **-0.159570** | -0.066370 |
| 8.10 | 0.703760 | 0.195556 | **1.019023** | 0.063438 |  | -0.068620 | -0.102220 | **-0.174810** | -0.079310 |
| 8.25 | 0.688887 | 0.167511 | **0.974376** | 0.043185 |  | -0.123760 | -0.122580 | **-0.181770** | -0.090110 |
| 8.40 | 0.720245 | 0.184274 | **1.063338** | 0.037256 |  | -0.114480 | -0.111600 | **-0.162480** | -0.086740 |
| 8.55 | 0.668570 | 0.151608 | **1.019056** | 0.062139 |  | -0.067980 | -0.109410 | **-0.168170** | -0.065340 |
| 8.70 | 0.704652 | 0.240365 | **0.908879** | 0.024232 |  | -0.098950 | -0.104520 | **-0.159450** | -0.086970 |
| 8.85 | 0.688280 | 0.161538 | **0.969511** | 0.018569 |  | -0.107720 | -0.102580 | **-0.186150** | -0.110040 |
| 9.00 | 0.668608 | 0.153574 | **0.967393** | 0.042186 |  | -0.099010 | -0.101300 | **-0.193980** | -0.079370 |
| 9.15 | 0.725879 | 0.134672 | **0.960145** | 0.013672 |  | -0.104790 | -0.095120 | **-0.210570** | -0.083870 |
| 9.30 | 0.708332 | 0.184913 | **0.953062** | 0.023299 |  | -0.128790 | -0.158110 | **-0.164380** | -0.102590 |
| 9.45 | 0.741549 | 0.162373 | **0.905338** | 0.004911 |  | -0.103070 | -0.133480 | **-0.190520** | -0.090690 |
| 9.60 | 0.678795 | 0.159842 | **0.896733** | 0.012440 |  | -0.125210 | -0.122310 | **-0.190270** | -0.089510 |
| 9.75 | 0.721194 | 0.180661 | **0.903187** | 0.025797 |  | -0.142520 | -0.103090 | **-0.148170** | -0.084550 |
| 9.90 | 0.707175 | 0.155860 | **0.894780** | -0.028800 |  | -0.146950 | -0.121580 | **-0.173360** | -0.129550 |
| 10.05 | 0.699605 | 0.150624 | **0.873202** | -0.014110 |  | -0.129280 | -0.107740 | **-0.178600** | -0.090620 |
| 10.20 | 0.703229 | 0.176654 | **0.867013** | 0.008076 |  | -0.121570 | -0.125280 | **-0.199010** | -0.067500 |
| 10.35 | 0.721535 | 0.153943 | **0.921224** | -0.033660 |  | -0.121540 | -0.094600 | **-0.160060** | -0.104740 |
| 10.50 | 0.745343 | 0.147749 | **0.866285** | -0.023940 |  | -0.175290 | -0.097650 | **-0.205040** | -0.095510 |
| 10.65 | 0.684524 | 0.197227 | **0.870058** | -0.016140 |  | -0.109760 | -0.091290 | **-0.174120** | -0.080090 |
| 10.80 | 0.698069 | 0.124816 | **0.813033** | -0.022040 |  | -0.142430 | -0.153620 | **-0.192820** | -0.095260 |
| 10.95 | 0.702072 | 0.150772 | **0.790329** | -0.003950 |  | -0.101790 | -0.116480 | **-0.174940** | -0.134980 |
| 11.10 | 0.732348 | 0.171468 | **0.824187** | -0.014710 |  | -0.164760 | -0.112700 | **-0.170970** | -0.064160 |
| 11.25 | 0.719505 | 0.132460 | **0.823227** | -0.021740 |  | -0.174000 | -0.092600 | **-0.163030** | -0.096350 |
| 11.40 | 0.695849 | 0.186855 | **0.843184** | -0.034160 |  | -0.131900 | -0.117450 | **-0.176710** | -0.075830 |
| 11.55 | 0.679497 | 0.148363 | **0.790131** | -0.048520 |  | -0.139180 | -0.133480 | **-0.182430** | -0.077240 |
| 11.70 | 0.651686 | 0.177097 | **0.752633** | -0.026400 |  | -0.114700 | -0.086880 | **-0.163780** | -0.102060 |
| 11.85 | 0.666009 | 0.181521 | **0.809889** | -0.015070 |  | -0.136250 | -0.131400 | **-0.153550** | -0.076220 |
| 12.00 | 0.623307 | 0.141849 | **0.824385** | -0.014110 |  | -0.119530 | -0.119930 | **-0.135890** | -0.064380 |
| 12.15 | 0.685435 | 0.139563 | **0.798504** | -0.048620 |  | -0.121850 | -0.142950 | **-0.168820** | -0.121710 |
| 12.30 | 0.692188 | 0.143496 | **0.761999** | -0.036960 |  | -0.142610 | -0.125510 | **-0.166740** | -0.075200 |
| 12.45 | 0.663315 | 0.140989 | **0.800093** | -0.043760 |  | -0.142130 | -0.134200 | **-0.155850** | -0.065070 |
| 12.60 | 0.662784 | 0.129437 | **0.787285** | -0.035060 |  | -0.097760 | -0.127430 | **-0.153040** | -0.090870 |
| 12.75 | 0.639925 | 0.087897 | **0.781029** | -0.038290 |  | -0.097270 | -0.124710 | **-0.164910** | -0.087870 |
| 12.90 | 0.653982 | 0.102719 | **0.749092** | 0.009142 |  | -0.177550 | -0.127210 | **-0.178930** | -0.058290 |
| 13.05 | 0.685795 | 0.152615 | **0.762562** | -0.055580 |  | -0.161750 | -0.132000 | **-0.176730** | -0.055000 |
| 13.20 | 0.683253 | 0.131526 | **0.752236** | -0.053720 |  | -0.151120 | -0.120990 | **-0.155920** | -0.049690 |
| 13.35 | 0.691372 | 0.125381 | **0.714970** | -0.063380 |  | -0.109210 | -0.118950 | **-0.170680** | -0.090220 |
| 13.50 | 0.674849 | 0.143963 | **0.685978** | -0.054910 |  | -0.134160 | -0.121860 | **-0.163230** | -0.089280 |
| 13.65 | 0.618033 | 0.092272 | **0.763422** | -0.049920 |  | -0.149770 | -0.151310 | **-0.153150** | -0.050230 |
| 13.80 | 0.665516 | 0.076861 | **0.662843** | -0.034760 |  | -0.172360 | -0.141640 | **-0.179810** | -0.078370 |
| 13.95 | 0.625014 | 0.064325 | **0.687401** | -0.080660 |  | -0.165810 | -0.119070 | **-0.163910** | -0.115150 |
| 14.10 | 0.612513 | 0.149690 | **0.698190** | -0.051220 |  | -0.159500 | -0.115520 | **-0.175300** | -0.087180 |
| 14.25 | 0.622377 | 0.114123 | **0.731054** | -0.064140 |  | -0.121590 | -0.140630 | **-0.136300** | -0.069930 |
| 14.40 | 0.603085 | 0.120096 | **0.650201** | -0.086090 |  | -0.117780 | -0.098960 | **-0.133550** | -0.077590 |
| 14.55 | 0.610388 | 0.090969 | **0.696039** | -0.079960 |  | -0.136750 | -0.129900 | **-0.184680** | -0.081590 |
| 14.70 | 0.592973 | 0.109330 | **0.674957** | -0.042690 |  | -0.135450 | -0.135150 | **-0.168920** | -0.106530 |
| 14.85 | 0.588895 | 0.115549 | **0.641563** | -0.063280 |  | -0.157250 | -0.159260 | **-0.167850** | -0.046310 |
| 15.00 | 0.578215 | 0.118622 | **0.663472** | -0.072270 |  | -0.124490 | -0.139400 | **-0.148960** | -0.106620 |
| 15.15 | 0.555754 | 0.097852 | **0.643813** | -0.087790 |  | -0.150870 | -0.122510 | **-0.146190** | -0.062470 |
| 15.30 | 0.589179 | 0.118769 | **0.668139** | -0.076170 |  | -0.127540 | -0.153890 | **-0.158300** | -0.025490 |
| 15.45 | 0.612247 | 0.064890 | **0.617535** | -0.048150 |  | -0.149990 | -0.122420 | **-0.146580** | -0.065370 |
| 15.60 | 0.591911 | 0.131673 | **0.657316** | -0.052620 |  | -0.145230 | -0.111890 | **-0.148520** | -0.030810 |
| 15.75 | 0.557328 | 0.085611 | **0.572591** | -0.059440 |  | -0.109950 | -0.081170 | **-0.178220** | -0.053310 |
| 15.90 | 0.601605 | 0.115967 | **0.644839** | -0.072300 |  | -0.152700 | -0.123150 | **-0.177700** | -0.079740 |
| 16.05 | 0.579884 | 0.115721 | **0.613233** | -0.100750 |  | -0.183270 | -0.124860 | **-0.204630** | -0.030600 |
| 16.20 | 0.590849 | 0.091436 | **0.602046** | -0.055110 |  | -0.129000 | -0.133380 | **-0.155350** | -0.074400 |
| 16.35 | 0.577778 | 0.106455 | **0.595063** | -0.115570 |  | -0.155750 | -0.148250 | **-0.171750** | -0.107130 |
| 16.50 | 0.576640 | 0.105865 | **0.612935** | -0.079300 |  | -0.158160 | -0.123130 | **-0.138240** | -0.069320 |
| 16.65 | 0.551296 | 0.060122 | **0.585961** | -0.070870 |  | -0.167200 | -0.124850 | **-0.171970** | -0.036480 |
| 16.80 | 0.513924 | 0.106405 | **0.622764** | -0.092920 |  | -0.108090 | -0.140060 | **-0.196680** | -0.061770 |
| 16.95 | 0.528759 | 0.113337 | **0.588179** | -0.100420 |  | -0.132300 | -0.115670 | **-0.171330** | -0.053280 |
| 17.10 | 0.488808 | 0.103726 | **0.589403** | -0.111080 |  | -0.162020 | -0.146340 | **-0.146300** | -0.073000 |
| 17.25 | 0.519217 | 0.122923 | **0.600822** | -0.099880 |  | -0.145980 | -0.148820 | **-0.130510** | -0.058250 |
| 17.40 | 0.493398 | 0.067987 | **0.584075** | -0.080760 |  | -0.115040 | -0.122100 | **-0.160530** | -0.067810 |
| 17.55 | 0.513640 | 0.089814 | **0.599034** | -0.082560 |  | -0.173840 | -0.143820 | **-0.135630** | -0.030940 |
| 17.70 | 0.497989 | 0.108863 | **0.600127** | -0.107010 |  | -0.108990 | -0.161200 | **-0.171180** | -0.067510 |
| 17.85 | 0.507816 | 0.093255 | **0.598869** | -0.140760 |  | -0.098910 | -0.142910 | **-0.151670** | -0.062330 |
| 18.00 | 0.495997 | 0.087356 | **0.515566** | -0.101750 |  | -0.122350 | -0.124440 | **-0.184170** | -0.049690 |
| 18.15 | 0.492583 | 0.119138 | **0.527448** | -0.108440 |  | -0.087120 | -0.120210 | **-0.143340** | -0.051110 |
| 18.30 | 0.444910 | 0.128011 | **0.556473** | -0.097820 |  | -0.107570 | -0.141020 | **-0.157660** | -0.023320 |
| 18.45 | 0.442254 | 0.105103 | **0.570770** | -0.140060 |  | -0.119820 | -0.127780 | **-0.148030** | -0.058980 |
| 18.60 | 0.498786 | 0.101317 | **0.585697** | -0.110440 |  | -0.115600 | -0.115820 | **-0.145810** | 0.007873 |
| 18.75 | 0.515650 | 0.041785 | **0.546743** | -0.108410 |  | -0.127730 | -0.144280 | **-0.164300** | -0.027040 |
| 18.90 | 0.495618 | 0.083546 | **0.508351** | -0.154010 |  | -0.125780 | -0.130690 | **-0.160740** | -0.022850 |
| 19.05 | 0.480707 | 0.086619 | **0.483033** | -0.094390 |  | -0.080400 | -0.127010 | **-0.180980** | -0.021270 |
| 19.20 | 0.469439 | 0.109773 | **0.494186** | -0.126070 |  | -0.077580 | -0.166260 | **-0.162080** | 0.001675 |
| 19.35 | 0.441647 | 0.093821 | **0.519670** | -0.166200 |  | -0.101420 | -0.138630 | **-0.184440** | -0.029040 |
| 19.50 | 0.439105 | 0.118277 | **0.513680** | -0.130100 |  | -0.164510 | -0.143420 | **-0.156610** | -0.025820 |
| 19.65 | 0.446693 | 0.067865 | **0.497297** | -0.137320 |  | -0.097270 | -0.142770 | **-0.151950** | -0.042830 |
| 19.80 | 0.419680 | 0.074919 | **0.500044** | -0.138760 |  | -0.114780 | -0.097960 | **-0.156740** | -0.023190 |
| 19.95 | 0.409132 | 0.096942 | **0.469265** | -0.164910 |  | -0.101790 | -0.119840 | **-0.160340** | -0.021680 |
| 20.10 | 0.389176 | 0.103456 | **0.557499** | -0.162240 |  | -0.116640 | -0.127100 | **-0.171130** | -0.040230 |
| 20.25 | 0.440357 | 0.075116 | **0.524138** | -0.160640 |  | -0.121630 | -0.117020 | **-0.156530** | -0.037450 |
| 20.40 | 0.431290 | 0.064006 | **0.543003** | -0.158310 |  | -0.119090 | -0.126620 | **-0.172120** | -0.020010 |
| 20.55 | 0.395986 | 0.024604 | **0.516493** | -0.130300 |  | -0.138070 | -0.146540 | **-0.172990** | -0.024170 |
| 20.70 | 0.364818 | 0.102989 | **0.500474** | -0.130400 |  | -0.152400 | -0.145790 | **-0.168690** | -0.002220 |
| 20.85 | 0.349129 | 0.073469 | **0.464003** | -0.147850 |  | -0.152690 | -0.138910 | **-0.172680** | -0.007420 |
| 21.00 | 0.371438 | 0.054297 | **0.459435** | -0.142290 |  | -0.127430 | -0.114800 | **-0.171600** | -0.010240 |
| 21.15 | 0.349091 | 0.054690 | **0.487104** | -0.140290 |  | -0.096230 | -0.164550 | **-0.178390** | 0.005236 |
| 21.30 | 0.343116 | 0.104685 | **0.476811** | -0.145090 |  | -0.134280 | -0.160900 | **-0.165980** | 0.002903 |
| 21.45 | 0.382821 | 0.078335 | **0.469563** | -0.167840 |  | -0.166120 | -0.128680 | **-0.172930** | 0.023499 |
| 21.60 | 0.388170 | 0.058672 | **0.472574** | -0.129330 |  | -0.091730 | -0.129640 | **-0.158780** | -0.012540 |
| 21.75 | 0.392268 | 0.073002 | **0.457913** | -0.111480 |  | -0.146890 | -0.124080 | **-0.155900** | 0.006755 |
| 21.90 | 0.364875 | 0.050167 | **0.408335** | -0.167340 |  | -0.127220 | -0.143700 | **-0.159440** | 0.007058 |
| 22.05 | 0.334124 | 0.032224 | **0.399366** | -0.131100 |  | -0.131510 | -0.138270 | **-0.146200** | 0.018129 |
| 22.20 | 0.369921 | 0.042646 | **0.436698** | -0.114740 |  | -0.103170 | -0.136160 | **-0.144570** | 0.001716 |
| 22.35 | 0.316273 | 0.055427 | **0.407475** | -0.129300 |  | -0.133020 | -0.158470 | **-0.161460** | 0.022257 |
| 22.50 | 0.321205 | 0.078016 | **0.375206** | -0.124830 |  | -0.120510 | -0.139300 | **-0.172990** | -0.014880 |
| 22.65 | 0.327294 | 0.086693 | **0.394699** | -0.121870 |  | -0.124460 | -0.164740 | **-0.173590** | 0.017342 |
| 22.80 | 0.279451 | 0.033453 | **0.394071** | -0.125030 |  | -0.130660 | -0.153960 | **-0.165270** | 0.056642 |
| 22.95 | 0.317107 | 0.038516 | **0.417304** | -0.091960 |  | -0.124660 | -0.131120 | **-0.140230** | -0.008150 |
| 23.10 | 0.330481 | 0.061203 | **0.340422** | -0.095220 |  | -0.101430 | -0.078560 | **-0.163920** | -0.008430 |
| 23.25 | 0.320408 | 0.058795 | **0.402047** | -0.092320 |  | -0.169090 | -0.163480 | **-0.136990** | 0.023596 |
| 23.40 | 0.321471 | 0.057615 | **0.386326** | -0.101120 |  | -0.142790 | -0.108690 | **-0.167720** | -0.006180 |
| 23.55 | 0.306579 | 0.068405 | **0.374147** | -0.111540 |  | -0.108570 | -0.131830 | **-0.137480** | -0.010320 |
| 23.70 | 0.283758 | 0.059827 | **0.351575** | -0.120070 |  | -0.112630 | -0.108950 | **-0.152690** | 0.059031 |
| 23.85 | 0.329799 | 0.067815 | **0.324668** | -0.100580 |  | -0.121070 | -0.115120 | **-0.125370** | -0.003530 |
| 24.00 | 0.269568 | 0.057492 | **0.303090** | -0.114570 |  | -0.132250 | -0.121950 | **-0.154590** | 0.017991 |
| 24.15 | 0.258053 | 0.058278 | **0.354719** | -0.100620 |  | -0.116820 | -0.121250 | **-0.178680** | -0.034060 |
| 24.30 | 0.288311 | 0.084136 | **0.315037** | -0.094850 |  | -0.113510 | -0.116360 | **-0.142890** | 0.022201 |
| 24.45 | 0.288500 | 0.064399 | **0.317023** | -0.098520 |  | -0.118340 | -0.128430 | **-0.138550** | -0.030760 |
| 24.60 | 0.239405 | 0.068921 | **0.318512** | -0.097750 |  | -0.145870 | -0.124220 | **-0.145190** | 0.007997 |
| 24.75 | 0.281348 | 0.075509 | **0.330725** | -0.101050 |  | -0.116120 | -0.117740 | **-0.143330** | 0.005623 |
| 24.90 | 0.301115 | 0.013052 | **0.334895** | -0.090860 |  | -0.129810 | -0.162770 | **-0.134700** | 0.018157 |
| 25.05 | 0.231722 | 0.025120 | **0.264930** | -0.100780 |  | -0.093850 | -0.139630 | **-0.135090** | -0.024350 |
| 25.20 | 0.273020 | 0.048938 | **0.317718** | -0.084860 |  | -0.074490 | -0.156180 | **-0.145480** | 0.063779 |
| 25.35 | 0.240411 | 0.024285 | **0.311595** | -0.114610 |  | -0.130510 | -0.133750 | **-0.134410** | -0.012460 |
| 25.50 | 0.208540 | 0.070740 | **0.251129** | -0.119340 |  | -0.130570 | -0.152000 | **-0.139650** | -0.015540 |
| 25.65 | 0.235383 | 0.077770 | **0.277837** | -0.138690 |  | -0.110240 | -0.132440 | **-0.131720** | 0.007666 |
| 25.80 | 0.200933 | 0.031536 | **0.280717** | -0.139420 |  | -0.134990 | -0.144890 | **-0.125790** | -0.028110 |
| 25.95 | 0.208844 | 0.033994 | **0.218298** | -0.106250 |  | -0.156600 | -0.142730 | **-0.127080** | -0.019600 |
| 26.10 | 0.243104 | 0.060982 | **0.278036** | -0.140460 |  | -0.144110 | -0.113810 | **-0.106450** | -0.016010 |

***used for representative trace in Figure 7I**
