# Supplementary material for: Neuronal junctophilins recruit specific CaV and RyR isoforms to ER-PM junctions and functionally alter CaV2.1 and CaV2.2
Source: eLife. 2021 Mar 26;10:e64249. doi: 10.7554/eLife.64249 (PMC8046434; doi:10.7554/eLife.64249)
Supplement: Figure 8—source data 1. [file elife-64249-fig8-data1.docx]

**Figure 8C RyR1, RyR2 & RyR3 vs Chimera “JPH3-with-JPH4 divergent**

(Pearson’s coefficients for RyR1, RyR2 & RyR3 vs JPH3 and JPH4 are in "Figure 5-source data 1”

**Pearson’s Coefficient**s

| **Cell** | **JPH3-with**-**JPH4 divergent vs** | | |
| --- | --- | --- | --- |
|  | **RyR1** | **RyR2** | **RyR3** |
| 1 | 0.36 | 0.37 | 0.38 |
| 2 | 0.34 | 0.48 | 0.31 |
| 3 | 0.27 | 0.28 | 0.41 |
| 4 | 0.39 | 0.29 | 0.54 |
| 5 | 0.32 | 0.19 | 0.41 |
| 6 | 0.21 | 0.18 | 0.77 |
| 7 | -0.15 | 0.19 | 0.3 |
| 8 | 0.38 | 0.26 | 0.57 |
| 9 | 0.11 | 0.17 | 0.39 |
| 10 | 0.14 | 0.18 | 0.38 |
| 11 | 0.42 | 0.19 | 0.38 |
| 12 | 0.12 | 0.07 | 0.72 |
| 13 | 0.22 | 0.14 | 0.29 |
| 14 | 0.3 | 0.16 | 0.26 |
| 15 | 0.3 | 0.22 | 0.15 |
| 16 | 0.17 |  | 0.21 |
| 17 | 0.07 |  |  |
| 18 | -0.01 |  |  |

**Statistical comparisons of {RyR1, RyR2 & RyR3 vs Chimera JPH3-with-JPH4-divergent} vs**

**{RyR1, RyR2 & RyR3 vs JPH3 and JPH4}**

(Statistical comparisons of JPH3 vs RyRs and JPH4 vs RyRs are reported in "Figure 5-source data 1”

**One-way ANOVA:** p < 0.0001

| **Tukey's multiple comparisons test** | **Mean Diff.** | **95% CI of diff.** | **Significant?** | **Summary** | **Adjusted p Value** |
| --- | --- | --- | --- | --- | --- |
|  |  |  |  |  |  |
| [JPH3 vs RyR1] vs [Chimera vs RyR1] | 0.5738 | 0.4613 to 0.6864 | Yes | **** | < 0.0001 |
| [JPH3 vs RyR1] vs [Chimera vs RyR2] | 0.5692 | 0.4502 to 0.6882 | Yes | **** | < 0.0001 |
| [JPH3 vs RyR1] vs [Chimera vs RyR3] | 0.3895 | 0.2728 to 0.5061 | Yes | **** | < 0.0001 |
| [JPH3 vs RyR2] vs [Chimera vs RyR1] | 0.2983 | 0.1827 to 0.4138 | Yes | **** | < 0.0001 |
| [JPH3 vs RyR2] vs [Chimera vs RyR2] | 0.2936 | 0.1718 to 0.4154 | Yes | **** | < 0.0001 |
| [JPH3 vs RyR2] vs [Chimera vs RyR3] | 0.1139 | -0.005611 to 0.2334 | No | ns | 0.0751 |
| [JPH3 vs RyR3] vs [Chimera vs RyR1] | 0.4855 | 0.3767 to 0.5943 | Yes | **** | < 0.0001 |
| [JPH3 vs RyR3] vs [Chimera vs RyR2] | 0.4808 | 0.3654 to 0.5963 | Yes | **** | < 0.0001 |
| [JPH3 vs RyR3] vs [Chimera vs RyR3] | 0.3011 | 0.1881 to 0.4141 | Yes | **** | < 0.0001 |
| [Chimera vs RyR1] vs [Chimera vs RyR2] | -0.004667 | -0.1330 to 0.1237 | No | ns | > 0.9999 |
| [Chimera vs RyR1] vs [Chimera vs RyR3] | -0.1844 | -0.3105 to -0.05825 | Yes | *** | 0.0003 |
| [Chimera vs RyR1] vs [JPH4 vs RyR1] | -0.1044 | -0.2305 to 0.02175 | No | ns | 0.1948 |
| [Chimera vs RyR1] vs [JPH4 vs RyR2] | -0.02727 | -0.1439 to 0.08939 | No | ns | 0.9982 |
| [Chimera vs RyR1] vs [JPH4 vs RyR3] | -0.3189 | -0.4298 to -0.2080 | Yes | **** | < 0.0001 |
| [Chimera vs RyR2] vs[ Chimera vs RyR3] | -0.1797 | -0.3116 to -0.04778 | Yes | *** | 0.0010 |
| [Chimera vs RyR2] vs [JPH4 vs RyR1] | -0.09971 | -0.2316 to 0.03222 | No | ns | 0.3056 |
| [Chimera vs RyR2] vs [JPH4 vs RyR2] | -0.02261 | -0.1455 to 0.1003 | No | ns | 0.9997 |
| [Chimera vs RyR2] vs [JPH4 vs RyR3] | -0.3143 | -0.4317 to -0.1968 | Yes | **** | < 0.0001 |
| [Chimera vs RyR3] vs [JPH4 vs RyR1] | 0.0800 | -0.04978 to 0.2098 | No | ns | 0.5906 |
| [Chimera vs RyR3] vs [JPH4 vs RyR2] | 0.1571 | 0.03650 to 0.2777 | Yes | ** | 0.0021 |
| [Chimera vs RyR3] vs [JPH4 vs RyR3] | -0.1346 | -0.2496 to -0.01952 | Yes | ** | 0.0094 |
