# Supplementary material for: Neuronal junctophilins recruit specific CaV and RyR isoforms to ER-PM junctions and functionally alter CaV2.1 and CaV2.2
Source: eLife. 2021 Mar 26;10:e64249. doi: 10.7554/eLife.64249 (PMC8046434; doi:10.7554/eLife.64249)
Supplement: Figure 8—figure supplement 1—source data 1. [file elife-64249-fig8-figsupp1-data1.docx]

**Figure 8-figure supplement 1A**

**Ca_V_1.2 vs JPH3-with-JPH4-divergent**

(Data for Ca_V_1.2 vs JPH3 and Ca_V_1.2 vs JPH4 are reported in “Figure 2-source data 1”)

**Pearson’s Coefficients**

| **Cell** | **Ca_V_1.2 vs**  **JPH3-with-JPH4-divergent** |
| --- | --- |
| 1 | 0.87 |
| 2 | 0.88 |
| 3 | 0.76 |
| 4 | 0.81 |
| 5 | 0.84 |
| 6 | 0.66 |
| 7 | 0.74 |
| 8 | 0.87 |
| 9 | 0.73 |
| 10 | 0.84 |
| 11 | 0.92 |
| 12 | 0.85 |
| 13 | 0.84 |
| 14 | 0.73 |
| 15 | 0.78 |

**Statistics**

**One-way ANOVA:** p < 0.0001

| **Tukey's multiple comparisons test** | **Mean Diff.** | **95% CI of diff.** | **Significant?** | **Summary** | **Adjusted p Value** |
| --- | --- | --- | --- | --- | --- |
|  |  |  |  |  |  |
| [Ca_V_1.2 vs JPH3] vs [Ca_V_1.2 vs JPH3-w-JPH4-divergent] | -0.07383 | -0.1421 to -0.005545 | Yes | * | 0.0311 |
| [Ca_V_1.2 vs JPH3] vs [Ca_V_1.2 vs JPH4] | 0.09217 | 0.03288 to 0.1515 | Yes | ** | 0.0012 |
| [Ca_V_1.2 vs JPH4] vs [Ca_V_1.2 vs JPH3-w-JPH4-divergent] | 0.1660 | 0.09824 to 0.2338 | Yes | **** | < 0.0001 |
