# Supplementary material for: Neuronal junctophilins recruit specific CaV and RyR isoforms to ER-PM junctions and functionally alter CaV2.1 and CaV2.2
Source: eLife. 2021 Mar 26;10:e64249. doi: 10.7554/eLife.64249 (PMC8046434; doi:10.7554/eLife.64249)
Supplement: Figure 8—figure supplement 1—source data 2. [file elife-64249-fig8-figsupp1-data2.docx]

**Figure 8-figure supplement 1B**

**I_peak_ and I_700_ for Ca_V_2.1 and Ca_V_2.2 with JPH3-with-JPH4-divergent**

(I_peak_ and I_700_ for Ca_V_1.2 and Ca_V_2.2 with JPH3 or JPH4 are reported in “Figure 3-source data 1”)

**Ca_V_2.1 + JPH3-with-JPH4-divergent**

**I_peak_ (pA/pF) I_700_ (pA/pF)**

| **Test Potential (mV)** | **cell 1** | **cell 2** | **cell 3** | **cell 4** | **cell 5** |
| --- | --- | --- | --- | --- | --- |
| 0 | -0.289 | -0.251 | -0.255 | -0.122 | -0.099 |
| 10 | -0.936 | -0.710 | -1.115 | -0.329 | -0.427 |
| 20 | -3.154 | -2.077 | -4.241 | -1.355 | -2.944 |
| 30 | -3.646 | -3.205 | -7.004 | -2.777 | -5.521 |
| 40 | -2.852 | -2.920 | -6.676 | -2.817 | -4.500 |
| 50 | -2.016 | -2.394 | -5.037 | -2.147 | -2.627 |
| 60 | -1.371 | -1.845 | -3.315 | -1.475 | -1.226 |
| 70 | -0.937 | -1.204 | -1.923 | -0.967 | * |

| **Test Potential (mV)** | **cell 1** | **cell 2** | **cell 3** | **cell 4** | **cell 5** |
| --- | --- | --- | --- | --- | --- |
| 0 | -0.552 | -0.511 | -0.481 | -0.396 | -0.258 |
| 10 | -1.637 | -1.722 | -1.646 | -0.678 | -0.748 |
| 20 | -6.261 | -6.985 | -8.160 | -2.612 | -4.029 |
| 30 | -12.265 | -15.684 | -19.671 | -8.133 | -9.814 |
| 40 | -12.171 | -15.832 | -19.962 | -10.076 | -10.200 |
| 50 | -8.871 | -11.246 | -14.162 | -7.723 | -7.144 |
| 60 | -5.638 | -6.926 | -8.652 | -4.942 | -4.500 |
| 70 | -3.192 | -3.902 | -4.818 | -2.732 | * |

* Cell died * Cell died

**CaV2.2 + JPH3-with-JPH4 divergent**

**I_peak_ (pA/pF)**

| **Test Potential (mV)** | **cell 1** | **cell 2** | **cell 3** | **cell 4** | **cell 5** | **cell 6** | **cell 7** | **cell 8** |
| --- | --- | --- | --- | --- | --- | --- | --- | --- |
| 0 | -1.634 | -1.828 | -1.761 | -1.154 | -1.800 | -1.430 | -1.092 | -1.560 |
| 10 | -7.012 | -9.699 | -11.351 | -6.415 | -9.751 | -6.856 | -5.454 | -9.983 |
| 20 | -83.130 | -46.936 | -58.689 | -28.914 | -40.553 | -29.083 | -22.852 | -73.914 |
| 30 | -108.690 | -80.713 | -85.194 | -51.261 | -60.346 | -58.183 | -45.743 | -119.024 |
| 40 | -81.360 | -68.632 | -67.342 | -44.833 | -49.161 | -53.439 | -43.064 | -93.520 |
| 50 | -59.205 | -49.495 | -46.502 | -31.709 | -34.305 | -38.269 | -31.635 | -65.978 |
| 60 | -41.794 | -32.213 | -29.796 | -20.459 | -21.887 | -25.433 | -20.492 | -44.000 |
| 70 | -27.406 | -18.745 | -17.395 | -12.048 | -13.243 | -15.499 | -12.096 | -26.921 |

**I_700_ (pA/pF)**

| **Test Potential (mV)** | **cell 1** | **cell 2** | **cell 3** | **cell 4** | **cell 5** | **cell 6** | **cell 7** | **cell 8** |
| --- | --- | --- | --- | --- | --- | --- | --- | --- |
| 0 | -0.531 | -0.845 | -0.982 | -0.599 | 2.513 | -0.593 | -0.613 | -0.718 |
| 10 | -1.998 | -4.202 | -5.390 | -3.173 | 2.630 | -2.671 | -2.789 | -2.884 |
| 20 | -7.640 | -14.024 | -17.005 | -9.698 | 1.721 | -8.464 | -9.024 | -9.226 |
| 30 | -14.527 | -18.240 | -20.908 | -12.594 | 1.226 | -12.779 | -12.116 | -14.878 |
| 40 | -14.848 | -15.903 | -17.309 | -11.523 | 1.113 | -12.530 | -10.487 | -16.042 |
| 50 | -13.648 | -12.284 | -13.288 | -9.220 | 1.034 | -10.611 | -8.224 | -14.896 |
| 60 | -11.666 | -9.323 | -9.487 | -6.548 | 0.795 | -8.012 | -5.837 | -11.828 |
| 70 | -8.529 | -5.760 | -5.983 | -4.266 | 0.141 | -5.258 | -3.837 | -7.125 |
