# Supplementary material for: Neuronal junctophilins recruit specific CaV and RyR isoforms to ER-PM junctions and functionally alter CaV2.1 and CaV2.2
Source: eLife. 2021 Mar 26;10:e64249. doi: 10.7554/eLife.64249 (PMC8046434; doi:10.7554/eLife.64249)
Supplement: Figure 9—source data 1. [file elife-64249-fig9-data1.docx]

**Figure 9D-F**

**RyR1_1:4300_, RyR2_1:4226_ and RyR3_1:4032_ vs JPH3 and JPH4**

**Pearson’s Coefficients**

| **Cell** | **RyR1_1:4300_ vs** | | **RyR2_1:4226_ vs** | | **RyR3_1:4032_ vs** | |
| --- | --- | --- | --- | --- | --- | --- |
|  | **JPH3** | **JPH4** | **JPH3** | **JPH4** | **JPH3** | **JPH4** |
| 1 | 0.64 | 0.15 | 0.08 | 0 | 0.71 | 0.05 |
| 2 | 0.77 | 0.11 | 0.34 | 0.12 | 0.84 | 0.42 |
| 3 | 0.69 | 0.14 | 0.16 | 0.12 | 0.73 | 0.35 |
| 4 | 0.46 | 0.17 | 0.26 | 0.08 | 0.8 | 0.13 |
| 5 | 0.85 | 0.25 | 0.31 | 0.3 | 0.78 | 0.13 |
| 6 | 0.53 | 0.29 | 0.23 | 0.07 | 0.88 | 0.4 |
| 7 | 0.82 | 0.2 | 0.2 | 0.46 | 0.9 | 0.36 |
| 8 | 0.86 | 0.24 | 0.01 | 0.38 | 0.88 | 0.14 |
| 9 | 0.77 | 0.41 | 0.33 | 0.36 | 0.82 | 0.25 |
| 10 | 0.89 | 0.3 | 0.27 | 0.16 | 0.83 | 0.37 |
| 11 | 0.23 | 0.28 | 0.1 | 0.24 | 0.91 |  |
| 12 | 0.77 | 0.43 | 0.1 | 0.29 | 0.87 |  |
| 13 | 0.57 | 0.47 |  | 0.16 | 0.84 |  |
| 14 | 0.23 |  |  | 0.22 | 0.71 |  |
| 15 | 0.77 |  |  |  | 0.82 |  |
| 16 | 0.66 |  |  |  | 0.84 |  |
| 17 | 0.78 |  |  |  | 0.88 |  |
| 18 | 0.11 |  |  |  | 0.85 |  |
| 19 | 0.92 |  |  |  | 0.79 |  |
| 20 | 0.87 |  |  |  | 0.82 |  |
| 21 | 0.85 |  |  |  | 0.81 |  |
| 22 | 0.82 |  |  |  | 0.88 |  |
| 23 | 0.68 |  |  |  | 0.77 |  |

**Statistics**

**One-way ANOVA:** p < 0.0001

| **Tukey's multiple comparisons test** | **Mean Diff.** | **95% CI of diff.** | **Significant?** | **Summary** | **Adjusted p Value** |
| --- | --- | --- | --- | --- | --- |
|  |  |  |  |  |  |
| [JPH3 vs RyR1_1:4300_] vs [JPH4 vs RyR1_1:4300_] | 0.4110 | 0.2640 to 0.5581 | Yes | **** | < 0.0001 |
| [JPH3 vs RyR1_1:4300_] vs [JPH3 vs RyR2_1:4226_] | 0.4765 | 0.3256 to 0.6274 | Yes | **** | < 0.0001 |
| [JPH3 vs RyR1_1:4300_] vs [JPH4 vs RyR2_1:4226_] | 0.4642 | 0.3206 to 0.6079 | Yes | **** | < 0.0001 |
| [JPH3 vs RyR1_1:4300_] vs [JPH3 vs RyR3_1:4032_] | -0.1487 | -0.2736 to -0.02374 | Yes | * | 0.0102 |
| [JPH3 vs RyR1_1:4300_] vs [JPH4 vs RyR3_1:4032_] | 0.4157 | 0.2551 to 0.5762 | Yes | **** | < 0.0001 |
| [JPH4 vs RyR1_1:4300_] vs [JPH3 vs RyR2_1:4226_] | 0.06545 | -0.1042 to 0.2351 | No | ns | 0.8702 |
| [JPH4 vs RyR1_1:4300_] vs [JPH4 vs RyR2_1:4226_] | 0.05319 | -0.1100 to 0.2164 | No | ns | 0.9323 |
| [JPH4 vs RyR1_1:4300_] vs [JPH3 vs RyR3_1:4032_] | -0.5597 | -0.7068 to -0.4127 | Yes | **** | < 0.0001 |
| [JPH4 vs RyR1_1:4300_] vs [JPH4 vs RyR3_1:4032_] | 0.004615 | -0.1736 to 0.1828 | No | ns | > 0.9999 |
| [JPH3 vs RyR2_1:4226_] vs [JPH4 vs RyR2_1:4226_] | -0.01226 | -0.1790 to 0.1544 | No | ns | > 0.9999 |
| [JPH3 vs RyR2_1:4226_] vs [JPH3 vs RyR3_1:4032_] | -0.6252 | -0.7761 to -0.4743 | Yes | **** | < 0.0001 |
| [JPH3 vs RyR2_1:4226_] vs [JPH4 vs RyR3_1:4032_] | -0.06083 | -0.2423 to 0.1206 | No | ns | 0.9242 |
| [JPH4 vs RyR2_1:4226_] vs [JPH3 vs RyR3_1:4032_] | -0.6129 | -0.7566 to -0.4693 | Yes | **** | < 0.0001 |
| [JPH4 vs RyR2_1:4226_] vs [JPH4 vs RyR3_1:4032_] | -0.04857 | -0.2240 to 0.1269 | No | ns | 0.9657 |
| [JPH3 vs RyR3_1:4032_] vs [JPH4 vs RyR3_1:4032_] | 0.5643 | 0.4038 to 0.7249 | Yes | **** | < 0.0001 |
