# Supplementary material for: Neuronal junctophilins recruit specific CaV and RyR isoforms to ER-PM junctions and functionally alter CaV2.1 and CaV2.2
Source: eLife. 2021 Mar 26;10:e64249. doi: 10.7554/eLife.64249 (PMC8046434; doi:10.7554/eLife.64249)
Supplement: Figure 10—source data 1. [file elife-64249-fig10-data1.docx]

**Figure 10F**

**RyR1_1:4300_ vs junctophilin constructs**

Pearson’s Coefficients

| **Cell** | **RyR1_1:4300_ vs** | | | | |
| --- | --- | --- | --- | --- | --- |
|  | **a** | **b** | **c** | **d** | **e** |
| 1 | 0.90 | 0.76 | 0.45 | 0.75 | 0.45 |
| 2 | 0.91 | 0.65 | 0.89 | 0.82 | 0.54 |
| 3 | 0.74 | 0.87 | 0.82 | 0.43 | 0.39 |
| 4 | 0.93 | 0.88 | 0.83 | 0.79 | 0.33 |
| 5 | 0.92 | 0.72 | 0.91 | 0.84 | 0.25 |
| 6 | 0.85 | 0.86 | 0.79 | 0.68 | 0.25 |
| 7 | 0.68 | 0.52 | 0.86 | 0.75 | 0.38 |
| 8 | 0.92 | 0.84 | 0.79 | 0.83 | 0.63 |
| 9 | 0.90 | 0.88 | 0.83 | 0.82 | 0.31 |
| 10 | 0.62 | 0.82 | 0.78 | 0.73 | 0.53 |
| 11 | 0.66 | 0.91 | 0.92 | 0.53 | 0.51 |
| 12 |  | 0.59 | 0.89 | 0.68 | 0.41 |
| 13 |  | 0.70 | 0.90 | 0.83 | 0.28 |
| 14 |  | 0.75 | 0.85 | 0.80 | 0.39 |
| 15 |  | 0.88 | 0.84 | 0.75 | 0.27 |
| 16 |  |  | 0.84 |  | 0.17 |
| 17 |  |  | 0.89 |  |  |
| 18 |  |  | 0.89 |  |  |
| 19 |  |  | 0.90 |  |  |
| 20 |  |  | 0.89 |  |  |
| 21 |  |  | 0.85 |  |  |
| 22 |  |  | 0.85 |  |  |

a: mCherry-JPH3(1-707)

b: mCherry-JPH4(1-414)-JPH3(418-707)

c: mCherry-JPH3 (418-748)

d: JPH3 (653-748)-mCherry

e: mCherry-ER

**Statistics**

One-way ANOVA: p < 0.0001

| **Tukey's multiple comparisons test** | **Mean Diff.** | **95% CI of diff.** | **Significant?** | **Summary** | **Adjusted p Value** |
| --- | --- | --- | --- | --- | --- |
|  |  |  |  |  |  |
| a vs. b | 0.04558 | -0.08151 to 0.1727 | No | ns | 0.8533 |
| a vs. c | -0.01818 | -0.1364 to 0.1000 | No | ns | 0.9927 |
| a vs. d | 0.08558 | -0.04151 to 0.2127 | No | ns | 0.3356 |
| a vs. e | 0.4403 | 0.3149 to 0.5657 | Yes | **** | < 0.0001 |
| b vs. c | -0.06376 | -0.1710 to 0.04344 | No | ns | 0.4629 |
| b vs. d | 0.0400 | -0.07690 to 0.1569 | No | ns | 0.8734 |
| b vs. e | 0.3947 | 0.2796 to 0.5098 | Yes | **** | < 0.0001 |
| c vs. d | 0.1038 | -0.003445 to 0.2110 | No | ns | 0.0625 |
| c vs. e | 0.4585 | 0.3533 to 0.5637 | Yes | **** | < 0.0001 |
| d vs. e | 0.3547 | 0.2396 to 0.4698 | Yes | **** | < 0.0001 |

a = RyR1_1:4300_ vs JPH3_1-707_

b = RyR1_1:4300_ vs JPH4_1-414_-JPH3_418-576_

c = RyR1_1:4300_ vs JPH3_418-748_

d = RyR1_1:4300_ vs JPH3_653-748_

e = RyR1_1:4300_ vs mCherry-ER
