# Supplementary material for: Neuronal junctophilins recruit specific CaV and RyR isoforms to ER-PM junctions and functionally alter CaV2.1 and CaV2.2
Source: eLife. 2021 Mar 26;10:e64249. doi: 10.7554/eLife.64249 (PMC8046434; doi:10.7554/eLife.64249)
Supplement: Figure 10—figure supplement 1—source data 1. [file elife-64249-fig10-figsupp1-data1.docx]

**Fig 10-figure supplement 1B**

**JPH3_Δ(681-725)_ vs RyR1**

(Data for JPH3 vs RyR1 in “Figure 5-source data 1”)

**Pearson’s Coefficients**

| **Cell** | **JPH3_Δ(681-725)_ vs RyR1** |
| --- | --- |
| 1 | 0.64 |
| 2 | 0.59 |
| 3 | 0.91 |
| 4 | 0.89 |
| 5 | 0.78 |
| 6 | 0.66 |
| 7 | 0.73 |
| 8 | 0.87 |
| 9 | 0.88 |
| 10 | 0.82 |
| 11 | 0.72 |
| 12 | 0.88 |
| 13 | 0.90 |
| 14 | 0.85 |
| 15 | 0.80 |
| 16 | 0.83 |
| 17 | 0.85 |
| 18 | 0.64 |
| 19 | 0.86 |
| 20 | 0.64 |
| 21 | 0.75 |
| 22 | 0.85 |
| 23 | 0.83 |

**Statistics**

[JPH3_Δ(681-725)_ vs RyR1] vs [JPH3 vs RyR1]

**T-test with Welch’s correction:** P = 0.9130
