# Supplementary material for: Neuronal junctophilins recruit specific CaV and RyR isoforms to ER-PM junctions and functionally alter CaV2.1 and CaV2.2
Source: eLife. 2021 Mar 26;10:e64249. doi: 10.7554/eLife.64249 (PMC8046434; doi:10.7554/eLife.64249)
Supplement: Figure 11—source data 1. [file elife-64249-fig11-data1.docx]

**Figure 11B**

**RyR3_1:4032_ vs JPH3(1-707)**

(Pearson’s coefficients for RyR3_1:4032_ vs JPH3 reported in ”Figure 9-source data 1”)

**Pearson’s Coefficients**

| **Cell** | **RyR3_1:4032_ vs JPH3(1-707)** |
| --- | --- |
| 1 | 0.45 |
| 2 | -0.09 |
| 3 | 0.40 |
| 4 | 0.10 |
| 5 | 0.16 |
| 6 | 0.09 |
| 7 | 0.34 |
| 8 | 0.38 |
| 9 | 0.22 |
| 10 | -0.09 |
| 11 | 0.14 |
| 12 | 0.27 |
| 13 | 0.08 |

**Statistics** (Fig 11B vs Fig 9F)

[RyR3_1:4032_ vs JPH3_1-707_] vs [RyR3_1:4032_ vs JPH3]

**T-test with Welch’s correction:** p < 0.0001
